# Reconstruction-aware radiomics for risk stratification of subsolid pulmonary nodules: technical evidence, clinical limits, and translation priorities

**DOI:** 10.3389/fmed.2026.1955926

**Published:** 2026-10-06

**Authors:** Yujian Liu, Yu Peng, Feng Chen, Jie Huang, Jie Yang, Jianquan Zhong

**Affiliations:** Department of Radiology, Zigong First People’s Hospital, Zigong, Sichuan, China

**Keywords:** clinical translation, CT reconstruction, invasive adenocarcinoma, part-solid nodule, pure ground-glass nodule, radiomics, subsolid pulmonary nodule, targeted reconstruction

## Abstract

Subsolid pulmonary nodules (SSNs), including pure ground-glass nodules (pGGNs) and part-solid nodules (PSNs), span transient inflammatory abnormalities and adenocarcinoma-spectrum lesions with widely different management implications. This critical narrative review treats CT reconstruction as part of the quantitative biomarker definition and asks whether targeted or high-spatial-resolution reconstruction provides stable, task-specific information beyond standardized thin-section CT. Evidence was synthesized across direct SSN reconstruction-domain studies, technical robustness studies, and clinical prediction studies. Direct imaging studies support improved depiction of selected fine structures, while same-scan GGN evidence demonstrates substantial pixel-size dependence of radiomic features and model composition. However, most clinical models were not evaluated in paired reconstruction domains, external validation with calibration remains uncommon, and no prospective study demonstrating management or patient benefit from reconstruction-aware radiomics in SSNs was identified in the literature reviewed. Persistent pGGNs provide a biological and technical rationale for investigating incremental quantitative information, but greater benefit than in PSNs has not been demonstrated. In PSNs, any model must add value beyond solid-component measurements, consolidation-to-tumor ratio, morphology, and longitudinal growth. Translation therefore requires reconstruction and segmentation robustness testing, strong conventional comparators, external calibration, prespecified thresholds, and prospective decision-linked evaluation. Until such benefit and safety are demonstrated, reconstruction-aware radiomics should remain investigational or confined to governed, domain-checked decision-support pathways.

## 1 Introduction

The expansion of low-dose CT screening has substantially increased detection of subsolid pulmonary nodules (SSNs), including pure ground-glass nodules (pGGNs) without a visible solid component and part-solid nodules (PSNs) containing both ground-glass and solid attenuation (1, 2). The clinical problem is no longer detection alone, but discrimination among transient inflammatory abnormalities, persistent indolent adenocarcinoma-spectrum lesions, and lesions with management-relevant invasive potential. Screening cohorts show that cancer probability and behavior differ between baseline and newly detected SSNs and according to the presence of a solid component (3–5). Misclassification has asymmetric consequences: overestimation may lead to repeated CT, anxiety, biopsy, or resection of indolent disease, whereas underestimation may delay treatment. Short-term stability is an imperfect endpoint because some persistent pGGNs grow only after several years (6–11), while completely resected adenocarcinoma *in situ* (AIS) and minimally invasive adenocarcinoma (MIA) have excellent long-term disease-specific survival (12).

Management relies on nodule type, total size, solid-component size, consolidation-to-tumor ratio (CTR), morphology, and temporal change. These variables are clinically powerful but technically vulnerable. Small solid foci may be obscured by partial-volume averaging, and apparent diameter, attenuation, volume, and solid-component boundaries vary with section thickness, reconstruction kernel, field of view (FOV), matrix size, dose, window settings, inspiratory level, and reader technique. Radiologic consolidation is also not identical to pathologic invasion: fibrosis, alveolar collapse, mucin, inflammation, and intralesional vessels may increase attenuation, while microscopic invasion may remain inconspicuous, particularly in a pGGN. Current Fleischner Society and American Association for Thoracic Surgery frameworks do not incorporate radiomics or reconstruction-domain compatibility into routine management algorithms (11, 13).

Targeted reconstruction and radiomics can be viewed as sequential components of one measurement chain. Changes in FOV, matrix, section thickness, kernel, or reconstruction algorithm can alter spatial sampling, partial-volume behavior, edge representation, noise, segmentation boundaries, and gray-level distributions before quantitative features are calculated (14–16). Improved visual sharpness is therefore not itself evidence of biomarker improvement; the relevant question is whether a reconstruction-defined image domain yields reproducible information that improves a prespecified clinical decision beyond standardized thin-section CT (Figure 1).

**Figure 1:**
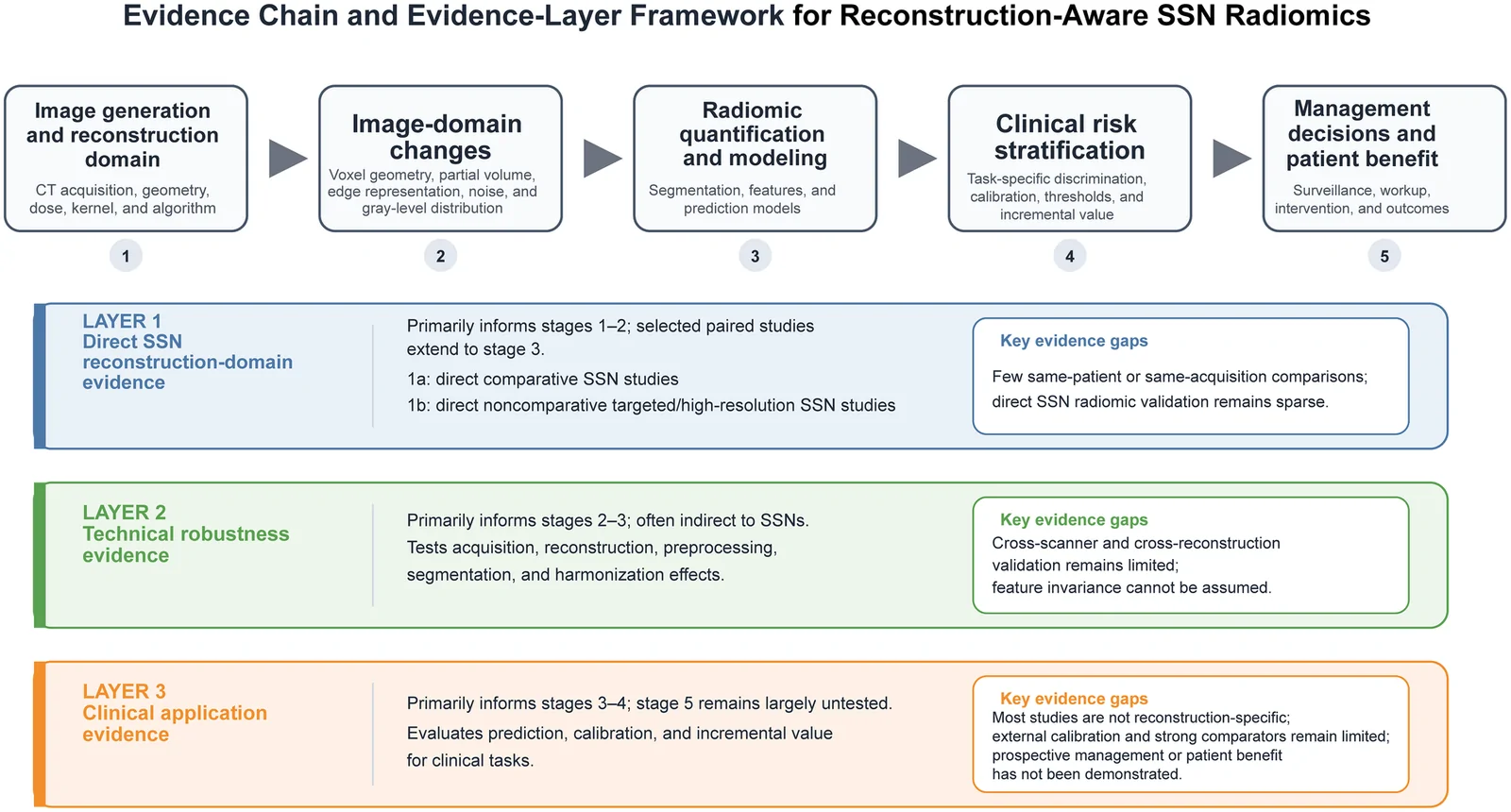
Evidence chain and author-proposed evidence-layer framework for reconstruction-aware radiomics in subsolid pulmonary nodules. The pathway progresses from image generation and image-domain change through radiomic quantification/modeling and clinical risk stratification to management decisions and patient benefit. Layer 1 comprises direct SSN reconstruction-domain evidence (Layer 1a, explicit comparative studies; Layer 1b, direct noncomparative targeted or high-spatial-resolution studies); Layer 2 comprises technical robustness evidence; and Layer 3 comprises clinical application evidence. Studies may contribute to more than one layer when they serve more than one evidentiary function. The framework is a qualitative organizational structure: evidentiary proximity does not represent methodological quality, formal certainty of evidence, consensus, or a clinical guideline. CT, computed tomography; SSN, subsolid pulmonary nodule.

This review focuses on pGGNs and PSNs and on clinically distinct tasks: confirmation of persistence, differentiation of adenocarcinoma-spectrum lesions from benign or transient abnormalities, estimation of pathologic invasiveness, longitudinal progression, and selected preoperative endpoints such as spread through air spaces (STAS). Prior reviews have generally addressed SSN prediction models or generic CT radiomic reproducibility as separate topics (7, 17–19); the present review focuses on their intersection with reconstruction domain. It follows the evidence chain from image generation and feature behavior to prediction, calibration, incremental value, and management-linked use, while separating direct SSN reconstruction evidence from indirect technical evidence.

The central premise is deliberately narrow: reconstruction settings define an applicability domain for quantitative biomarkers, but reconstruction-aware radiomics is clinically relevant only if domain-specific information remains stable and adds value beyond conventional CT, longitudinal assessment, and expert interpretation. pGGNs and PSNs are therefore treated as different prediction problems rather than as a single SSN category.

## 2 Methods

A critical narrative review design was selected because the literature spans CT acquisition and reconstruction, feature reproducibility, prediction modeling, and clinical management and is too heterogeneous for meaningful quantitative pooling. The review was developed with reference to the six quality domains of the Scale for the Assessment of Narrative Review Articles (SANRA): justification of importance, statement of aims, description of literature searching, appropriate referencing, scientific reasoning, and presentation of clinically relevant endpoint data. SANRA informed methodological self-assessment but was not used as a reporting certification, formal quality grade, study-level risk-of-bias instrument, or evidence-certainty framework (20).

Database searches of PubMed/MEDLINE, Embase, Web of Science Core Collection, and Scopus were completed on June 30, 2026. Three complementary modules addressed: (1) direct targeted or high-spatial-resolution SSN imaging and reconstruction-domain evidence; (2) clinical radiomics and quantitative-imaging applications; and (3) technical robustness, reproducibility, and harmonization. Terms within concept blocks were combined with OR and blocks within each module with AND using database-specific syntax. No language or document-type restrictions were applied. Complete database-specific implementations and search strings are provided in Supplementary Table 1 and Supplementary Methods M1-M4; the SANRA-informed self-assessment and proposed reconstruction-domain reporting items are provided in Supplementary Tables 2, 3.

Eligibility was defined according to evidentiary function. Direct SSN studies were included when they addressed targeted or high-spatial-resolution imaging, reconstruction-domain variation, or quantitative prediction relevant to persistence, malignancy, pathologic invasiveness, growth, STAS, or management. Broader pulmonary-nodule studies were included when they informed acquisition- or reconstruction-dependent robustness, segmentation, calibration, or transportability. Phantom and nonpulmonary technical studies were used only to inform technical sensitivity or reproducibility and not as evidence of SSN biological validity or clinical utility. Relevant standards, reporting guidance, systematic reviews, and methodological studies were also considered. Duplicate reports or studies without an interpretable link to the reconstruction-radiomics evidence chain were not prioritized.

Study selection was purposeful and interpretive rather than exhaustive. One author performed primary screening and evidence selection, and two additional authors verified retained records and evidence-layer classification; disagreements were resolved through discussion. Representative studies were selected to capture distinct evidence functions, prioritizing directness to the SSN reconstruction question, isolation of acquisition or reconstruction effects, independent or external evaluation, clinically meaningful comparators or endpoints, calibration or decision relevance, and major credibility or applicability concerns. When several studies served a similar role, the more methodologically informative or more recent study was selected. The overall workflow is shown in Figure 2, and standardized extraction fields are provided in Supplementary Table 4. “Not reported in the current review extraction” indicates that an item was not captured in the narrative evidence record, not necessarily that it was absent from the source publication.

**Figure 2:**
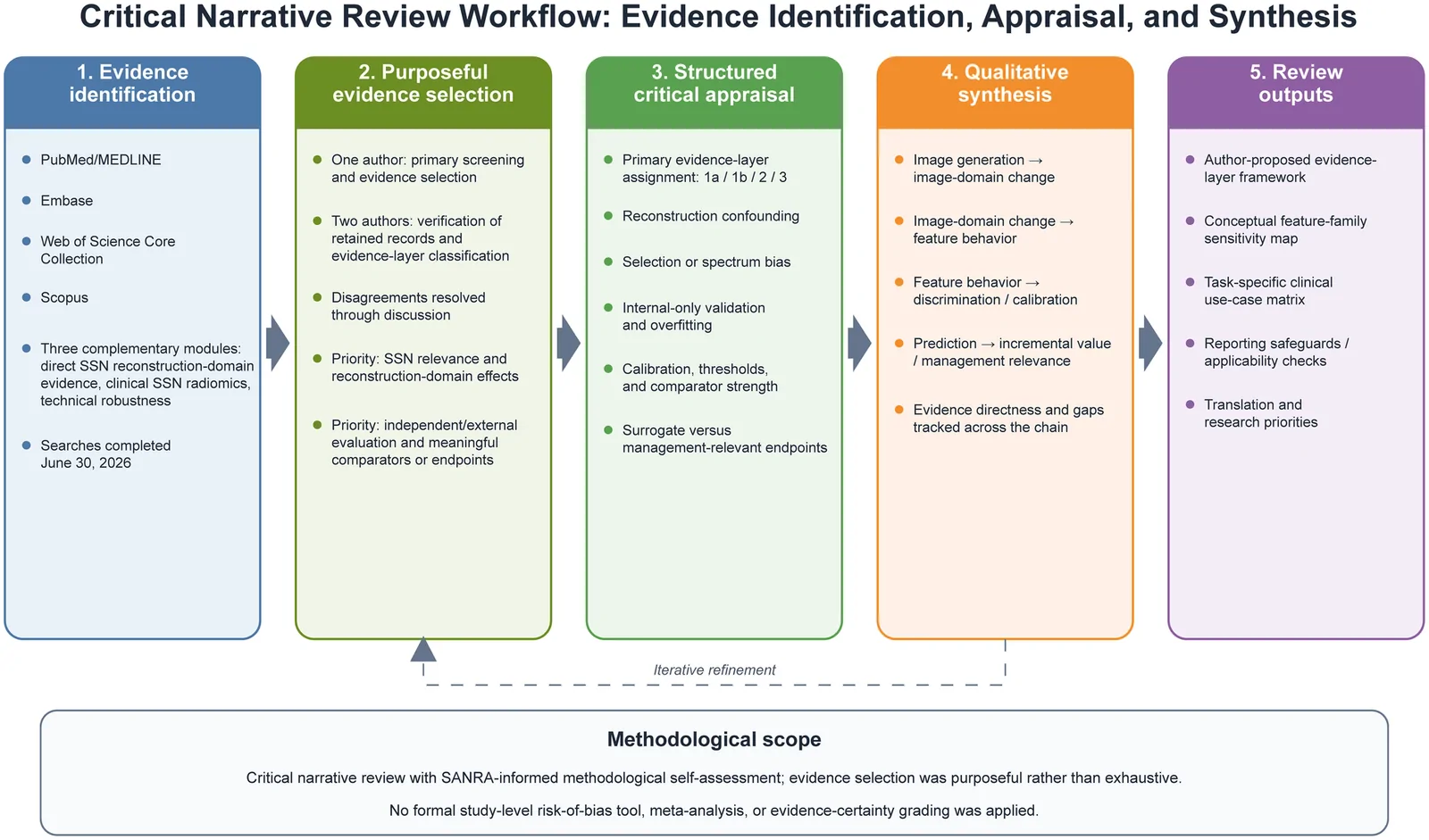
Critical narrative review workflow. Evidence was identified in PubMed/MEDLINE, Embase, Web of Science Core Collection, and Scopus using three complementary modules addressing direct SSN reconstruction-domain evidence, clinical SSN radiomics, and technical robustness evidence. One author performed primary screening and evidence selection, two additional authors verified retained records and evidence-layer classification, and disagreements were resolved through discussion. Purposeful selection emphasized SSN relevance, reconstruction-domain effects, independent or external evaluation, and clinically meaningful comparators or endpoints. Critical appraisal considered evidence function, reconstruction confounding, selection or spectrum concerns, validation, calibration, thresholds, comparator strength, and management relevance before qualitative synthesis. The dashed pathway denotes iterative refinement during synthesis. SANRA informed methodological self-assessment; no formal study-level risk-of-bias tool, meta-analysis, or evidence-certainty grading was applied. SANRA, scale for the assessment of narrative review articles; SSN, subsolid pulmonary nodule.

The author-proposed evidence-layer framework organizes studies by evidentiary function and proximity to the clinical question. Layer 1a comprises direct SSN studies with explicit imaging- or reconstruction-domain comparisons; Layer 1b comprises direct SSN studies using targeted, ultra-high-resolution, or other high-spatial-resolution techniques without a standard-domain comparator; Layer 2 comprises technical robustness studies addressing acquisition, reconstruction, preprocessing, segmentation, or harmonization; and Layer 3 comprises clinical application studies addressing decision-relevant outcomes. Primary assignment yielded 36 Layer 1a, 19 Layer 1b, 236 Layer 2, and 167 Layer 3 records (458 total). These are evidence-inventory counts rather than systematic-review inclusion totals or certainty grades. Studies addressing more than one evidentiary function could contribute conceptually to multiple layers, although one primary layer was used for counting. Evidence-layer proximity does not imply methodological quality, risk of bias, or certainty.

Design terminology was defined operationally. “Paired” denotes matched observations from the same patient or lesion; “same-patient” allows separate acquisitions in the same individual; “same-scan” refers to images derived from the same CT examination; and “same-acquisition” is reserved for different reconstructions generated from the same raw projection data. “Comparative” denotes any explicit domain comparison and does not necessarily imply patient matching or a shared acquisition. Same-acquisition paired reconstructions therefore provide the closest isolation of reconstruction-specific effects.

Critical appraisal considered reconstruction confounding, selection or spectrum bias, internal-only validation, overfitting, incomplete calibration, surrogate endpoints, weak comparators, and absent clinical-impact evaluation. Evidence was interpreted according to function: technical or phantom studies can establish sensitivity or reproducibility mechanisms but not SSN biological validity, whereas pathology-confirmed surgical cohorts may support discrimination while yielding biased absolute risks for screening populations.

Figure 3 is an author-developed conceptual sensitivity map rather than an evidence-grade matrix. H indicates repeatedly reported and comparatively prominent feature-family dependence on the technical factor; M indicates a recurrent but more moderate or context-dependent pattern; U indicates sparse, inconsistent, or insufficient evidence; and N/A = not directly applicable. No universal numerical threshold or voting rule was imposed because studies used heterogeneous perturbations, feature implementations, and stability metrics. Discordant or sparse evidence was assigned U or qualified in the text. The map was not independently scored by two raters and has not undergone external validation; supporting studies are summarized in Supplementary Table 5. The evidence layers, use-case matrix, safeguards, and reporting items are likewise author-developed qualitative synthesis outputs rather than certainty grades, consensus statements, reporting standards, or clinical guidelines.

**Figure 3:**
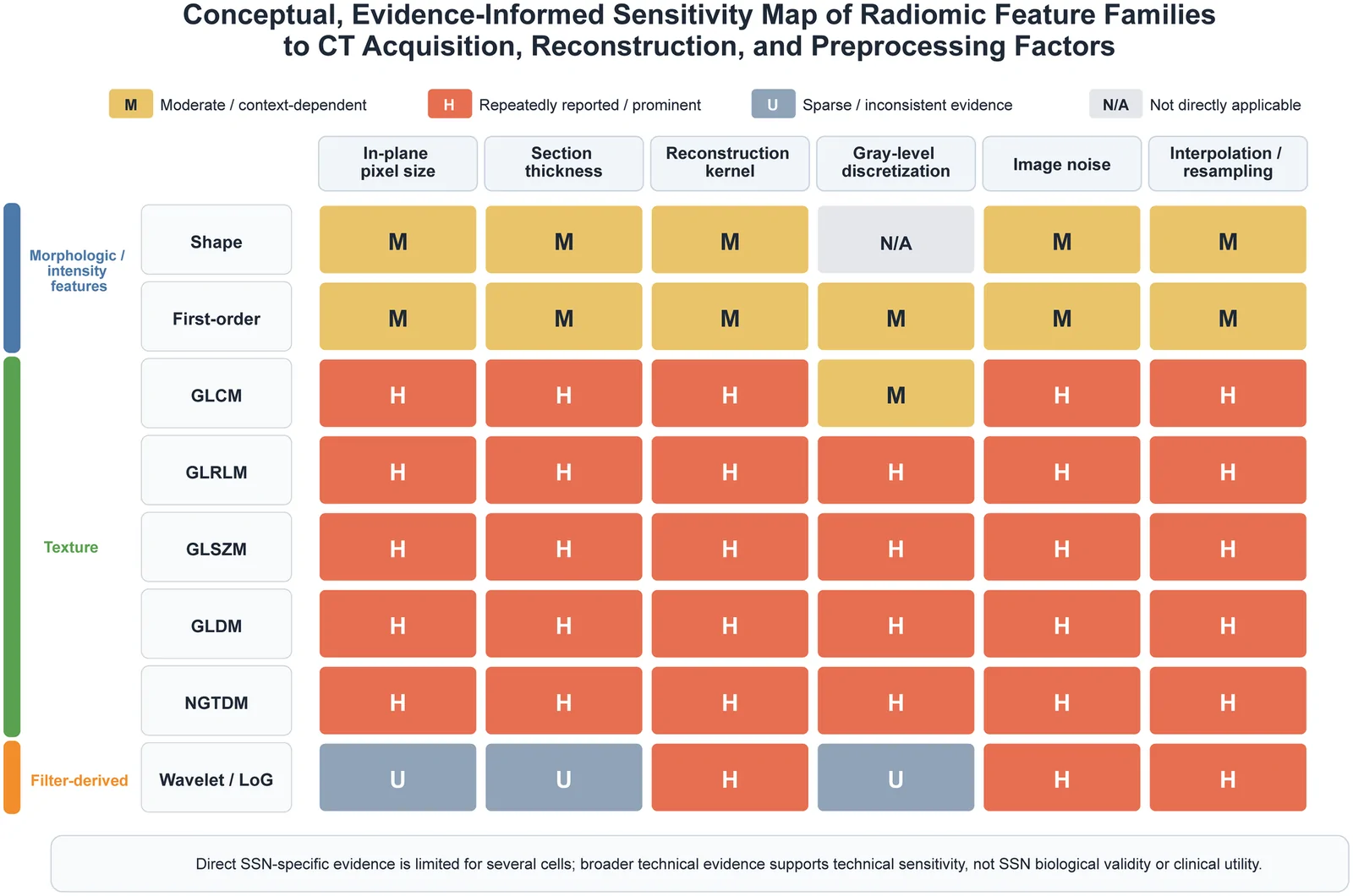
Conceptual, evidence-informed sensitivity map of radiomic feature families to CT acquisition, reconstruction, and preprocessing factors. M denotes a recurrent but moderate or context-dependent sensitivity pattern; H denotes repeatedly reported and comparatively prominent dependence; U denotes insufficient, sparse, or inconsistent evidence; and N/A = not directly applicable. These author-developed labels are descriptive synthesis categories, not numerical thresholds, pooled effect estimates, formal certainty grades, or invariant properties of individual features. Sensitivity may vary across protocols, scanners, segmentation methods, and preprocessing pipelines. Direct SSN-specific evidence is limited for several assignments; broader pulmonary-nodule and technical radiomics evidence was used only to inform technical sensitivity, not SSN biological validity or clinical utility. The map has not undergone external validation and should not be interpreted as a consensus statement or clinical recommendation. Primary supporting references and feature-family assignments are summarized in Supplementary Table 5. CT, computed tomography; GLCM, gray-level co-occurrence matrix; GLRLM, gray-level run-length matrix; GLSZM, gray-level size-zone matrix; GLDM, gray-level dependence matrix; NGTDM, neighborhood gray-tone difference matrix; LoG, laplacian-of-Gaussian.

## 3 Clinical and pathologic context

An SSN is a CT phenotype rather than a pathologic diagnosis. A pGGN is a focal area of increased attenuation through which pulmonary vessels and bronchial structures remain visible without a definite solid component; a PSN contains both ground-glass and solid attenuation. Newly detected SSNs may be transient, whereas persistence increases the probability of an adenocarcinoma-spectrum lesion. Solitary and multifocal SSNs also represent different management contexts: solitary lesions are usually assessed independently, whereas multifocal disease requires lesion-level prioritization and patient-level consideration of cumulative surveillance and treatment burden. In multifocal disease, the prediction unit should therefore be explicit. A lesion-level probability describes an individual nodule and should not be interpreted as an independent patient-level risk estimate; conversely, patient-level management must integrate the number, distribution, behavior, and treatment implications of multiple lesions.

Transient inflammation, focal fibrosis, hemorrhage, and organizing pneumonia can produce an SSN appearance. Persistent lesions may represent atypical adenomatous hyperplasia (AAH), AIS, MIA, or invasive adenocarcinoma (IAC), and these categories should not be collapsed into a single malignant class. AAH is a recognized precursor lesion, AIS shows lepidic growth without stromal, vascular, or pleural invasion, and MIA contains an invasive component measuring no more than 5 mm (21–23). AIS and MIA have near-zero recurrence after complete resection, whereas IAC behavior depends on invasive size, grade, lymphovascular or pleural invasion, STAS, and nodal status (12, 22–25). The 2021 World Health Organization classification incorporates the International Association for the Study of Lung Cancer grading framework for invasive adenocarcinoma (22, 23, 25).

Most radiomics studies address binary invasiveness tasks rather than aggressive histologic patterns and use inconsistent groupings, including AAH/AIS versus MIA/IAC, AIS/MIA versus IAC, MIA versus IAC, or AAH labeled as benign. These tasks are not interchangeable because they correspond to different clinical questions and error costs. A model that distinguishes preinvasive lesions from MIA/IAC may not be suitable for deciding whether a persistent lesion can continue surveillance, and a model trained on a pathology-confirmed surgical cohort cannot automatically estimate risk in a screening population.

The presence and size of a solid component in a PSN are among the strongest conventional markers of invasive risk. Nevertheless, radiologic consolidation is not a direct measurement of pathologic invasive size. Fibrosis, collapse, mucin, inflammation, and vascular structures may increase attenuation, while microscopic invasion may remain inconspicuous. Solid-component assessment should distinguish diameter, volume, proportion, attenuation, and longitudinal growth. For PSNs, a radiomics model must demonstrate incremental value over these strong baseline variables rather than reproduce them in a more complex form. For pGGNs, the absence of a visible solid component provides a biological and technical rationale for investigating subtle heterogeneity, boundary complexity, and vessel relationships as potential sources of additional information; however, direct comparative evidence has not established that reconstruction-aware radiomics provides greater incremental clinical value in pGGNs than in PSNs.

Prediction tasks should be separated according to the intended decision. Transient-versus-persistent classification addresses the need for short-interval confirmation; non-neoplastic-versus-adenocarcinoma-spectrum classification addresses continued observation or further workup; AIS/MIA versus IAC, or MIA versus IAC, addresses pathologic invasiveness and possible timing of intervention; and future growth, increasing mass, or solid transformation addresses surveillance intensity. STAS and high-grade patterns are extended endpoints relevant to operative planning. pGGN and PSN evidence should be analyzed separately because baseline risk, measurable features, segmentation behavior, and technical stability differ (Table 1).

**Table 1:** Clinical intended-use matrix for reconstruction-aware radiomics in subsolid pulmonary nodules.

Clinical task	Unresolved clinical problem	Conventional/longitudinal CT baseline	Potential reconstruction-aware contribution	Intended output/action	Error consequences and evidence required
Newly detected SSN	Transient/reactive opacity versus persistent lesion.	SSN type, size, morphology, clinical context/guideline category; short-interval CT when indicated.	Attenuation, boundary, and texture may refine persistence risk; management benefit remains unproven.	Calibrated persistence risk with uncertainty; routine versus short-interval review.	FP: excess CT/work-up/anxiety; FN: delayed confirmation. Evidence: screening/diagnostic cohorts, explicit persistence definition, external calibration, threshold-linked harms.
Persistent pGGN	Management-relevant invasive risk without a visible solid-component predictor.	Size, volume, mass, attenuation, morphology, longitudinal change, expert assessment.	Texture, shape, attenuation, and vessel-related features may refine risk; greater value than in PSNs is unproven.	Calibrated IAC risk; surveillance versus multidisciplinary review.	FP: premature biopsy/surgery; FN: delayed IAC treatment. Evidence: pGGN-specific external validation, strong comparators, calibration, net benefit.
PSN with uncertain invasive risk	Added information beyond an already informative solid component.	Solid-component diameter/volume, CTR, morphology, total size, longitudinal growth.	Whole-lesion, solid, ground-glass, or interface features are useful only if incremental to strong conventional/longitudinal predictors.	Calibrated incremental IAC risk; timing of surgical or multidisciplinary review.	FP: overtreatment; FN: delayed invasive-disease assessment. Evidence: direct comparison with solid-component metrics, CTR, morphology/growth, external calibration, thresholds, net benefit.
Longitudinal surveillance	Biological progression versus measurement or reconstruction drift.	Serial total/solid-component size, volume, mass, attenuation, morphology, and interval change under matched protocols.	Longitudinal/delta features may quantify change only when acquisition, reconstruction, segmentation, and timing are sufficiently consistent.	Probability of meaningful progression; maintain, shorten, or escalate surveillance.	FP: technical drift labeled progression; FN: missed progression. Evidence: long follow-up, protocol consistency, patient-level validation, calibration, action-linked thresholds.
Multifocal SSNs	Lesion prioritization within cumulative patient-level surveillance/treatment burden.	Lesion morphology/growth plus distribution, multiplicity, comorbidity, and treatment feasibility.	Lesion-level ranking may aid prioritization; nodule probabilities are not independent patient-level risks.	Identify lesions needing closer review within a patient-level plan.	FP: cumulative over-surveillance/procedures; FN: under-prioritization of the dominant lesion. Evidence: patient-level splitting, cluster-aware validation, lesion- versus patient-level endpoints.
Preoperative assessment	Planning support without converting a pathology surrogate into automatic resection extent.	Anatomy/location, CTR, margin feasibility, nodal assessment, lung function, comorbidity, operative factors, patient preference.	Intratumoral/regional/peritumoral features may support IAC/STAS risk estimation but should not determine surgery independently.	Supportive risk estimate for multidisciplinary planning, with uncertainty/abstention.	FP: excessive resection/intervention; FN: inadequate aggressive-disease planning. Evidence: prospective clinical-impact evaluation with surgical, functional, and patient-relevant outcomes.

This author-developed matrix is qualitative, not a validated clinical pathway or consensus recommendation. Tasks are not interchangeable; FP/FN consequences refer to proposed intended use, not validated action thresholds. Multifocality and background structural lung disease may modify applicability.

AIS, adenocarcinoma *in situ*; CTR, consolidation-to-tumor ratio; FP, false positive; FN, false negative; IAC, invasive adenocarcinoma; MIA, minimally invasive adenocarcinoma; pGGN, pure ground-glass nodule; PSN, part-solid nodule; SSN, subsolid pulmonary nodule; STAS, spread through air spaces.

## 4 Reconstruction as an image-domain intervention

Conventional thin-section CT depicts most SSNs but may not optimally characterize the smallest solid foci, subtle heterogeneity, or complex margins. Partial-volume averaging reduces apparent contrast and affects attenuation and volumetric measurements. In pGGNs, low contrast and vessel inclusion complicate segmentation; in PSNs, solid-component measurements vary with section thickness, kernel, window settings, and reader technique. Longitudinal comparison is weakened when scanner or reconstruction protocols change, even when the apparent visual difference is subtle.

For terminology consistency, this review distinguishes targeted scanning, targeted reconstruction, ultra-high-resolution acquisition, and image magnification as separate interventions. Targeted scanning acquires new projection data over a limited range and adds radiation. Targeted reconstruction uses existing raw data and may combine a smaller display FOV, larger matrix, smaller in-plane pixels, thin sections, and a high-spatial-frequency kernel, but the achievable geometry remains constrained by the original acquisition. Ultra-high-resolution CT improves spatial resolution through detector and acquisition design (26), whereas image magnification changes display scale only. These terms are not used interchangeably. Study-specific expressions such as “target scan” or “target reconstruction” are retained only when describing terminology used in the source report.

Direct SSN evidence remains limited. Zhu et al. compared a new ultra-high-resolution target scan with conventional target reconstruction in 91 patients with ground-glass-nodule-like lung adenocarcinoma and reported improved depiction of selected fine structural signs and greater agreement with pathologic categories (27). The study supports improved morphologic representation but changed both acquisition and reconstruction and did not evaluate radiomic stability, calibration, or management impact. Ren et al. evaluated 306 pathology-confirmed persistent pGGNs in 260 patients using a dedicated ultra-high-resolution target-scanning protocol; larger diameter and higher representative attenuation were independently associated with invasive pathology (28). This demonstrated the feasibility of ultra-high-resolution morphologic risk assessment but did not provide a paired comparison against a standardized thin-section reconstruction or establish incremental value over that baseline.

For PSNs, targeted reconstruction is biologically and technically plausible because improved edge representation may affect measurement of the solid component and the ground-glass/solid interface. However, a reconstruction-induced boundary shift can make quantitative comparison with a standardized thin-section reconstruction non-equivalent. Direct evidence therefore supports improved depiction and hypothesis generation, but not claims of superior clinical outcomes or automatic transfer of models between reconstruction domains.

The potential benefit is constrained by a resolution-noise-stability trade-off (Table 2). Smaller pixels and thinner sections can reduce partial-volume averaging and improve edge representation, but fewer photons per voxel increase noise. Sharp kernels preserve high-frequency structure while amplifying high-frequency noise. Iterative and deep-learning reconstruction can reduce noise but may modify edge sharpness and texture (29, 30). In a same-scan study of 182 patients with ground-glass nodules, 87% of 788 radiomic features varied across pixel sizes; smaller-pixel models performed better internally, but selected feature sets differed markedly across domains (31). This demonstrates domain dependence rather than universal superiority of the smallest pixel size.

**Table 2:** Reconstruction parameters, evidence directness, and task-specific implications for subsolid pulmonary nodule radiomics.

Parameter/approach	Imaging effect	Radiomic implication	Evidence directness	pGGN-specific implication	PSN-specific implication
Reconstructed field of view, matrix, and pixel size	Denser sampling and less in-plane partial-volume averaging; noise may increase.	Shifts segmentation, shape, first-order, texture, filtered features, and model composition.	Direct same-scan GGN pixel-size evidence plus broader technical studies (31–34).	May better depict low-contrast margins/heterogeneity but increase noise and segmentation instability.	Can shift the ground-glass/solid interface, solid-component measurements, and CTR; added benefit is unproven.
Section thickness and reconstruction interval	Thinner sections reduce through-plane averaging but may increase noise; shorter intervals add overlap, not resolution.	Can alter segmentation, feature values, and selected signatures, especially in small nodules.	Mainly mixed-nodule, phantom, and robustness evidence; limited paired SSN evidence (17, 32–34).	Affects subtle high-attenuation foci and whole-lesion attenuation distributions.	Affects solid-focus size and interface; longitudinal comparison requires matched settings.
Reconstruction kernel	Sharper kernels improve edges but increase high-frequency noise.	Texture/filtered features are strongly kernel-dependent; boundary features may shift.	Mainly mixed-nodule and technical reproducibility evidence (17, 33, 34).	May convert noise into apparent heterogeneity or small high-attenuation foci.	Can alter solid-component boundaries/interface texture and distort conventional information.
Radiation dose and image noise	Lower dose increases noise and may reduce low-contrast conspicuity.	Reduces segmentation stability/feature repeatability and may alter threshold performance.	Same-patient and technical lung-nodule evidence; limited direct SSN radiomic evidence (29, 30, 33, 34).	Low contrast/small size make pGGNs vulnerable to noise-driven texture and boundary variation.	May affect residual ground-glass/interface; longitudinal dose matching remains important.
Iterative, model-based, or deep-learning reconstruction	Noise reduction with algorithm-dependent changes in edge sharpness/noise texture.	Denoising/smoothing can shift feature distributions, signatures, and calibration.	Chest CT/GGN image-quality evidence; limited direct paired SSN radiomic validation (29, 30).	May improve visibility while suppressing subtle heterogeneity; report/test algorithm and strength.	Can alter solid-component edges/interface; compare locked algorithm levels.
Ultra-high-resolution acquisition	Improves spatial sampling and depiction of fine structures/minute solid foci.	Defines a distinct acquisition domain, not equivalent to targeted reconstruction from conventional raw data.	Direct SSN morphologic and emerging high-resolution model evidence; limited paired radiomic validation (27, 28, 41–44).	May improve internal architecture/focal-density depiction; quantitative gain is unproven.	May improve minute solid-focus/interface measurement; superiority over thin-section CT is unproven.
Photon-counting CT	Enhanced spatial/spectral sampling with lower electronic noise in selected modes.	Defines a domain dependent on thickness, kernel, energy, and reconstruction/denoising.	Emerging simulation, phantom, morphologic, and same-day characterization evidence (35–41, 44).	Potential low-contrast/spectral advantage; reproducible radiomic benefit is unproven.	May improve solid-component/spectral characterization; full acquisition-reconstruction chain defines applicability.

Evidence directness reflects the proximity of the cited evidence to the SSN-specific reconstruction question and should not be interpreted as a formal evidence grade or certainty assessment. Task-specific implications are evidence-informed interpretations rather than validated clinical recommendations.

CT, computed tomography; CTR, consolidation-to-tumor ratio; GGN, ground-glass nodule; pGGN, pure ground-glass nodule; PSN, part-solid nodule; SSN, subsolid pulmonary nodule.

Indirect technical evidence from broader pulmonary-nodule, nonpulmonary, and phantom settings shows that section thickness, kernel, dose, voxel geometry, scanner, and their interactions are major sources of feature variability, with small nodules particularly vulnerable (17, 32–34). Photon-counting CT and other high-spatial-resolution systems improve sampling and may reduce electronic noise, but quantitative outputs remain dependent on section thickness, pitch, energy settings, and the reconstruction chain (35–44). Better visual quality, more accurate measurement, and more stable radiomic features are distinct endpoints and should be evaluated separately.

## 5 Reconstruction-dependent radiomic information

Shape features quantify volume, surface area, compactness, sphericity, elongation, and contour complexity. Smaller voxels may better represent subtle lobulation or irregular margins, particularly in small nodules (31), but they also increase sensitivity to segmentation. A one-voxel boundary displacement represents a large proportional change in a 5-mm lesion, and segmentation algorithms trained on standard reconstructions may not behave identically on targeted images. These considerations support reconstruction and segmentation perturbation testing before outcome-based feature selection (17, 31–34).

First-order features describe attenuation distributions, including mean, median, percentiles, variance, skewness, kurtosis, energy, and entropy. Reducing voxel size may separate low-density lepidic regions from minute higher-attenuation foci, thereby changing the histogram. Texture features derived from gray-level co-occurrence, run-length, size-zone, dependence, and neighborhood gray-tone matrices quantify spatial relationships and are sensitive to voxel dimensions, kernel, noise, interpolation, and gray-level discretization (32, 34). High-frequency texture may contain genuine heterogeneity, reconstruction noise, or both. Filtered wavelet and Laplacian-of-Gaussian features introduce additional parameter dependence (45). Figure 3 provides a qualitative feature-family sensitivity map; its ratings are not pooled effect estimates or universal properties of individual features.

For pGGNs, whole-lesion three-dimensional segmentation is often used as the primary strategy because no stable solid subregion is visible. For PSNs, whole-lesion, ground-glass, solid, interface, and peritumoral analyses may provide different information, but the boundary between ground-glass and solid tissue is reconstruction-dependent. Peritumoral features may capture vessels, stroma, inflammation, or aeration changes, yet are easily contaminated by pleura, bronchi, fissures, or emphysema. A study comparing 2- and 5-mm peritumoral regions did not establish a universal margin (46). Habitat regions are best described as computational subregions unless spatial pathologic validation is available.

Reconstruction-aware preprocessing aims to standardize computation without obscuring the source image domain. Resampling can harmonize nominal voxel dimensions but cannot restore information lost through thick sections or smoothing, and interpolation itself changes texture. A fixed bin width can be appropriate for CT, but the bin width and intensity range need prespecification and sensitivity testing in low-contrast pGGNs. Image Biomarker Standardisation Initiative (IBSI) definitions and reference values and open implementations such as PyRadiomics support computational consistency (45, 47, 48). ComBat, CovBat, and related harmonization methods can reduce batch effects (49–55), but they cannot recover missing spatial information and may suppress biological variation when center and outcome are associated. Harmonization is therefore an evaluated modeling intervention, not a guarantee of transportability.

All preprocessing, harmonization, stability filtering, feature selection, and tuning must be confined to the training data and repeated within resampling loops. Related nodules, time points, and reconstructions from the same patient must remain in the same partition. Technical stability should precede outcome-based selection and include segmentation and reconstruction perturbation, reader or algorithm agreement, and cross-scanner testing. No feature family should be presumed universally reconstruction-invariant without empirical testing: small SSNs can show large proportional changes after a one-voxel boundary shift, while texture and filtered features are often parameter-dependent. Identical patient-level partitions and prespecified metrics are required when models are compared across reconstruction domains (Table 3).

**Table 3:** Major methodological risks and minimum safeguards for reconstruction-aware radiomics.

Methodological issue	Consequence	Minimum safeguard
Reconstruction-domain heterogeneity	Pixel size, thickness, kernel, dose, or algorithm can shift segmentation/features and limit transportability.	Lock/model the domain; use paired and cross-domain robustness testing.
Segmentation instability	Small boundary changes disproportionately affect pGGN and small-PSN features.	Report reader/algorithm agreement; use perturbation testing, QC, and failure criteria.
Patient-level leakage and pseudoreplication	Related nodules, time points, reconstructions, or derived images crossing partitions inflate performance.	Split by patient; keep related data together; nest preprocessing/feature selection.
High-dimensional overfitting and model-selection bias	Large searches relative to sample size yield unstable signatures and optimistic estimates.	Use prespecified robustness filtering, penalization, nested validation, optimism correction, and external validation.
Endpoint and label heterogeneity	Persistence, growth, STAS, and alternative pathology groupings are clinically non-equivalent.	Define task/reference standard; report task- and subgroup-specific performance.
Weak comparators	Complex models may appear useful against inadequate baselines.	Compare with guidelines, experts, size, solid-component measures, CTR, volume, mass, and attenuation as appropriate.
Incomplete performance evaluation	AUC alone does not establish probability accuracy, threshold safety, or clinical benefit.	Report CIs, calibration, Brier score, threshold metrics, decision curves, and failure analysis.
Spectrum and surgery bias	Resection cohorts overrepresent malignancy/IAC and underrepresent transient or benign SSNs.	Use intended-use screening/diagnostic cohorts and independent external validation.
Threshold transport and calibration drift	The same score may imply different risks/actions across sites, populations, or reconstruction domains.	Evaluate external calibration/local thresholds; prespecify updating and post-deployment monitoring.
Workflow, governance, and economic barriers	Manual processing, incompatibility, unclear accountability, cost, and unsupported updates threaten adoption/safety.	Use automated QC, interoperability, audit/version control, human oversight, and prospective workflow/economic evaluation.

AUC, area under the receiver operating characteristic curve; CTR, consolidation-to-tumor ratio; IAC, invasive adenocarcinoma; pGGN, pure ground-glass nodule; PSN, part-solid nodule; SSN, subsolid pulmonary nodule; STAS, spread through air spaces.

Reconstruction-domain model comparisons should use identical patient-level partitions and prespecified metrics and should report discrimination, calibration, threshold performance, and decision-curve analysis when an action threshold is proposed (56–58). Comparators should reflect the intended task and include guideline category, expert assessment, total and solid-component size, CTR, volume, mass, attenuation, morphology, and longitudinal change as appropriate. Existing radiomics and prediction-model reporting frameworks provide complementary safeguards (14, 18, 47, 59–63), but none substitutes for reconstruction-specific validation.

Deep learning has shown competitive performance for pulmonary nodule malignancy and SSN invasiveness (19, 64–72), but learned representations remain vulnerable to reconstruction-domain shift. Multi-domain training, reconstruction-aware augmentation, domain generalization, and adaptation are candidate strategies (73, 74). These methods cannot restore source spatial information that was not preserved by acquisition or reconstruction, and adaptation may alter calibration or create silent failure modes. Evaluation therefore benefits from holding out complete reconstruction domains or centers, comparison with a locked no-adaptation baseline, and reporting of calibration and abstention or failure-detection behavior. Current paired evidence does not establish that targeted reconstruction preferentially benefits handcrafted radiomics, deep learning, or vision-language models.

## 6 Clinical evidence and applications

Clinical SSN prediction evidence needs to be interpreted separately from reconstruction-specific evidence because most radiomics or artificial-intelligence models were not developed as paired reconstruction-domain comparisons (7, 9, 10, 75). For translation, six domains need to be distinguished: image depiction or measurement; feature robustness; discrimination; calibration and threshold performance; incremental value over strong conventional predictors or expert assessment; and prospective management or patient benefit. Evidence at one domain does not establish the next. General pulmonary-nodule, phantom, and nonpulmonary studies can inform technical sensitivity but not SSN-specific biological validity, while surgery-enriched pathology cohorts may inflate apparent absolute risk relative to screening populations (76).

Persistent pGGNs represent a hypothesis-generating application because no visible solid component provides a comparably dominant conventional predictor. Radiomics and deep learning may quantify subtle attenuation heterogeneity, contour complexity, vessel relationships, and peritumoral changes (46, 65, 67, 70, 71, 77). A 2026 texture-analysis study of 298 resected SSNs further supported the feasibility of distinguishing MIA from IAC, but its retrospective pathology-confirmed design and lack of external validation limit population-level risk estimation (78). Direct comparative evidence has not established that reconstruction-aware radiomics adds more value in pGGNs than in PSNs; pGGN-focused use therefore remains a rationale for further study rather than a demonstrated clinical advantage. Prediction tasks must remain explicit: AAH/AIS versus MIA/IAC, AIS/MIA versus IAC, and MIA versus IAC answer different questions (Table 1). Models intended to support surveillance need high sensitivity for management-relevant IAC, calibrated absolute risk, and explicit consideration of delayed-treatment harm, with pGGN-specific comparison against size, mass, attenuation, longitudinal change, and expert interpretation.

For PSNs, solid-component diameter, volume, proportion, CTR, morphology, and interval growth are strong baseline predictors and should constitute the minimum conventional comparison set whenever the data are available. Regional radiomics may characterize the solid component, residual ground-glass component, and interface, but any clinical value must be shown to be incremental rather than a more complex representation of the visible solid component. In a multicenter study of 297 PSN adenocarcinomas, a model separating solid and ground-glass radiomic features outperformed Brock, clinical-semantic, volumetric, and whole-lesion models in a held-out test set and showed good calibration (79). This does not show that targeted reconstruction caused the gain. More recent regional models support feasibility (80) but still require comparison with solid-component diameter or volume, CTR, morphology, and longitudinal growth before reconstruction-aware radiomics can be claimed to improve decision making.

Combined models integrate clinical factors, lesion measurements, semantic morphology, and radiomics (77, 79, 80). Multicenter studies better expose case-mix and protocol heterogeneity than single-center internal splits, but fusion is justified only when each component adds stable information and the final model is compared with a strong clinical-imaging baseline. Studies in pure-solid stage I lung cancer or survival prediction (81, 82) are methodologically relevant but not endpoint-matched to SSN invasiveness. Incremental value should be assessed through prediction error, calibration, net benefit, and clinically interpretable reclassification at prespecified thresholds.

External validation is most informative when it preserves the intended clinical setting rather than repeating testing in another surgery-enriched sample. Absolute risk and predictive values depend on prevalence and case mix; thresholds derived in high-prevalence pathology cohorts can yield excessive false positives when transferred directly to screening populations. Calibration-in-the-large, calibration slope, and threshold performance need evaluation before and after local recalibration. Reader comparisons also need to specify access to longitudinal imaging, clinical data, and guideline categories because these determine comparator strength.

Longitudinal models may align more closely with management because they address surveillance timing. Baseline and delta-radiomics have been evaluated for GGN growth or invasive pathology (10, 75, 83). A 2026 multicenter study combined baseline, follow-up, and delta-radiomic features with clinical-semantic variables and reported external validation, calibration, and locked three-tier thresholds (84). This strengthens the case for decision-linked longitudinal models but does not establish reconstruction-specific benefit or prospective management impact. Valid analyses still require sufficient follow-up, consistent reconstruction, patient-level splitting, and appropriate handling of multiple nodules; delta features become difficult to interpret biologically when acquisition or reconstruction changes are not controlled or explicitly modeled. Retrospective STAS models (85, 86) remain pathology-based intermediate endpoints, and no prospective study has shown that model-guided resection improves outcomes. Prognostic associations after sublobar resection (24) do not validate a model-informed surgical strategy.

Table 4 and Supplementary Table 4 summarize representative evidence. Direct SSN reconstruction-domain studies mainly support improved depiction, morphologic characterization, or domain-dependent feature behavior; indirect pulmonary-nodule, phantom, and nonpulmonary studies support technical sensitivity and robustness but not SSN biological validity. Clinical SSN prediction studies provide varying evidence for discrimination, calibration, or incremental performance, yet most do not isolate reconstruction-specific benefit. None of the representative studies demonstrates prospective management or patient benefit from reconstruction-aware radiomics. The evidence is therefore strongest for technical feasibility and domain sensitivity, intermediate for task-specific prediction, and weakest for reconstruction-specific incremental clinical value. The most informative next design is a same-patient, preferably same-acquisition, comparison of standardized thin-section and targeted or high-spatial-resolution reconstruction that assesses segmentation, feature drift, discrimination, calibration, threshold stability, net benefit, workflow, and patient-relevant outcomes.

**Table 4:** Representative evidence matrix: directness, design, validation, and translational meaning.

Study	Evidence source/design	Population/task	Reconstruction or analytic comparison	Validation/calibration/clinical comparator	Translational interpretation
Zhu et al. 2019 (27)	Direct SSN; Layer 1a; comparative imaging-domain study	91 patients; GGN-like adenocarcinoma; morphology-pathology	Ultra-high-resolution target scan versus conventional target reconstruction; acquisition and reconstruction both changed.	Pathology correlation; no radiomic validation/calibration or management comparator captured.	Better fine-structure depiction/pathologic agreement; reconstruction-specific radiomic gain and clinical benefit unproven.
Ren et al. 2021 (28)	Direct SSN; Layer 1b; noncomparative high-resolution study	260 patients/306 persistent pGGNs; preinvasive versus invasive pathology	Dedicated ultra-high-resolution target scan; no paired standard-domain comparator.	Pathology reference; no external validation/calibration captured.	High-resolution morphologic risk assessment feasible; reconstruction-attributable incremental value unproven.
Tao et al. 2021 (31)	Direct GGN; Layers 1a/2; same-scan pixel-size comparison	182 patients; GGN invasiveness signatures	Same-scan pixel-size domains; features/signatures changed across domains.	Internal evaluation; no external validation/calibration, locked action threshold, or strong clinical comparator captured.	Demonstrates domain dependence; smaller-pixel superiority and clinical utility unproven.
Meyer et al. 2019 (33)	Indirect non-SSN technical; Layer 2; within-patient reconstruction study	78 patients/151 liver metastases; 28 reconstructions; reproducibility	Dose/reconstruction settings varied; section thickness had a major effect.	Technical reproducibility endpoint; no SSN calibration/comparator.	Strong technical-sensitivity evidence; limited relevance to SSN biology/management.
Emaminejad et al. 2021 (34)	Indirect pulmonary-nodule technical; Layer 2; multivariable parameter study	89 screening patients with solid nodules >4 mm; 36 technical conditions	Dose, thickness, and kernel varied; simulated dose reduction included.	Technical reproducibility; no SSN calibration, incremental comparator, or management endpoint.	Shows parameter interactions/instability; no SSN validity or clinical-impact evidence.
Hertel et al. 2023 (36)	Indirect phantom technical; Layer 2; photon-counting CT test-retest	Organic phantoms; 104 features; position/dose repeatability	Photon-counting CT test-retest, repositioning, and dose conditions.	Repeatability only; no patient calibration, clinical comparator, or SSN endpoint.	Technical repeatability under defined conditions; not biological/clinical validation.
Wu et al. 2020 (79)	Clinical SSN, not reconstruction-specific; Layer 3; multicenter prediction	291 patients/297 PSN adenocarcinomas; held-out test	Regional solid/ground-glass radiomics; no targeted-reconstruction comparison.	Held-out test; calibration; Brock, clinical-semantic, volumetric, whole-lesion comparators.	PSN discrimination/calibration and incremental performance supported; reconstruction-specific gain/management benefit unproven.
Wu et al. 2021 (46)	Clinical GGN, not reconstruction-specific; Layer 3; regional/peritumoral radiomics	121 GGN adenocarcinomas; 2- versus 5-mm peritumoral regions	Peritumoral-margin comparison; no reconstruction-domain comparison.	Single-center internal study; no external validation; no universal margin.	Peritumoral-feature feasibility supported; incremental/reconstruction-specific value unproven.
Xue et al. 2024 (83)	Clinical longitudinal GGN, not reconstruction-specific; Layer 3; delta-radiomics	303 resected GGNs; baseline/follow-up; non-IAC versus IAC	Baseline/follow-up/delta features; heterogeneous scanners/matrices; no controlled reconstruction comparison.	Internal 7:3 split; no external validation captured; delta model not significantly better than conventional radiomics-clinical model.	Longitudinal feasibility supported; no clear incremental gain from added complexity.
Bao et al. 2025 (86)	Clinical SSN-related, not reconstruction-specific; Layer 3; multicenter STAS prediction	Three-center retrospective surgical cohort; STAS	Intratumoral/peritumoral/clinical model; no reconstruction-domain comparison.	External test cohort; calibration and decision-curve analysis reported.	Externally tested pathology-surrogate prediction; prospective surgical/patient benefit unproven.

Evidence source labels distinguish direct SSN reconstruction-domain, clinical SSN (not reconstruction-specific), indirect pulmonary-nodule technical, and phantom/nonpulmonary technical evidence. Layer 1a = direct comparative SSN; Layer 1b = direct noncomparative targeted/high-resolution SSN; Layer 2 = technical robustness; Layer 3 = clinical application. Layers indicate function/proximity, not methodological quality or certainty. Validation, calibration, comparator, and endpoint fields reflect the current review extraction; Supplementary Table 4 provides expanded standardized extraction.

## 7 Discussion

### 7.1 Clinical meaning and intended use

The current evidence separates technical effect from clinical consequence. Direct SSN and same-scan studies show that reconstruction can change depiction, measurements, feature values, and model composition, while clinical SSN studies show that radiomics can discriminate selected outcomes. These findings answer different questions. The unresolved translational issue is whether a reconstruction-defined image domain provides calibrated, incremental information that changes a clinically meaningful decision rather than merely producing a sharper image or a different feature set.

Intended use should be anchored to the clinical decision rather than to model architecture. Table 1 separates persistence confirmation, invasive-risk refinement, longitudinal progression, multifocal prioritization, and preoperative support, each with different comparators and error costs. Quantitative modeling is most defensible when conventional and longitudinal CT leave residual uncertainty that could change a specific action. False reassurance can delay appropriate treatment, whereas false-positive classification can intensify surveillance, trigger invasive work-up, or precipitate premature surgery.

The distinction between pGGNs and PSNs remains clinically important, but the proposed differential role of radiomics is still hypothesis-generating. The absence of a dominant visible solid component in pGGNs provides a rationale for studying subtle heterogeneity, boundary complexity, and vessel relationships; in PSNs, any added value must exceed the information already provided by solid-component measurements and longitudinal change. Likewise, prediction of IAC or STAS should not be mapped directly to resection extent: operative decisions still require integration of lesion location, CTR, margin feasibility, nodal assessment, lung function, comorbidity, operative expertise, and patient preference (87–90).

Accordingly, reconstruction-aware radiomics should currently be evaluated as a governed, second-layer risk-refinement approach within guideline-based assessment rather than as an autonomous diagnostic pathway. A clinically usable output would need calibrated absolute risk or prespecified risk categories with uncertainty, confirmation that the input lies within the supported reconstruction domain, and a predefined link to actions such as routine surveillance, shorter-interval review, or multidisciplinary discussion. A higher area under the receiver operating characteristic curve (AUC) alone is insufficient; the relevant test is whether the model improves threshold-specific decisions over conventional imaging and expert assessment without unacceptable harm.

### 7.2 Context dependence and longitudinal assessment

Longitudinal use is clinically attractive but technically vulnerable. Serial volume, mass, attenuation, solid-component measurements, and delta-radiomic features are most interpretable when acquisition, reconstruction, segmentation, and preprocessing are sufficiently comparable; otherwise domain drift can mimic progression or obscure true change. Model output should be interpreted with prior CT examinations and clinical context, and changes in scanner, kernel, section thickness, dose, or reconstruction algorithm warrant compatibility checks before a quantitative trajectory is used for management. Multimodal information may be useful in selected patients, but current evidence does not show that multimodal fusion resolves reconstruction-related instability.

Patient and pulmonary-disease context further define transportability. Emphysema commonly coexists with nodules in screening populations (91), while nodules in fibrosing interstitial lung disease are interpreted against distorted, heterogeneous parenchyma and often require longitudinal, multimodal assessment (92). These studies do not directly test reconstruction-aware radiomics, so they cannot establish model degradation. Nevertheless, emphysematous spaces, reticulation, traction bronchiectasis, architectural distortion, heterogeneous attenuation, and irregular nodule-parenchyma interfaces could plausibly affect segmentation, peritumoral sampling, attenuation distributions, and texture. Background structural lung disease should therefore be prespecified as an applicability domain, with subgroup performance, calibration, and clinically relevant interactions examined before transportability is assumed.

Multifocal SSNs and incomplete reconstruction provenance require similar caution. Lesion-level predictions from multiple nodules in one patient are correlated and must not be treated as independent patient-level risks; related nodules, time points, and reconstructions should remain within the same data partition. In deployment, missing or incomplete DICOM reconstruction metadata, vendor-specific kernel nomenclature, proprietary iterative or deep-learning reconstruction algorithms, and software-version changes can create an unrecognized domain mismatch. When compatibility cannot be established, the safer response is abstention or expert review rather than forced model output. These constraints make acquisition and reconstruction provenance part of the model specification rather than optional reporting detail.

### 7.3 Requirements for clinical translation and remaining limitations

Evidence development should progress from isolating reconstruction effects to testing transportability and, ultimately, clinical impact. Paired same-patient studies, preferably same-acquisition reconstructions from the same raw data, can quantify changes in segmentation, conventional measurements, feature drift, and model predictions. Multicenter studies should then test locked models and thresholds across vendors, kernels, doses, populations, and clinically relevant lung-disease contexts, with external calibration and explicit domain-shift analysis rather than discrimination alone. Prospective impact studies are ultimately needed to determine whether model-supported decisions alter surveillance, biopsy, or surgery appropriately and reduce unnecessary intervention without delaying treatment of invasive disease. pGGNs and PSNs can be modeled separately or assessed with prespecified interactions, while multifocal and longitudinal cohorts require patient-level partitioning.

Clinical deployment would additionally require reliable segmentation, automated quality control, reconstruction-domain checks, interpretable uncertainty, local recalibration rules, version control, and a predefined response to unsupported inputs. Post-deployment monitoring should track segmentation failure, abstention, calibration drift, threshold-specific errors, and performance across scanners, vendors, doses, reconstruction algorithms, and patient subgroups. Any material change in acquisition, reconstruction, segmentation, or feature extraction should trigger verification because it can alter the effective model even when the user interface remains unchanged.

Workflow and economic consequences are part of clinical utility rather than downstream implementation details. High-spatial-resolution reconstruction can increase image volume, storage, transfer, processing, and quality-control workload, while false-positive or false-negative predictions can change downstream testing and treatment. Prospective evaluations should therefore measure processing time, clinician override, follow-up intensity, invasive procedures, delayed diagnosis, stage at treatment, complications, quality of life, and pathway cost. Current economic evidence remains sparse and largely model-based (93, 94); CHEERS-AI provides relevant reporting guidance for future evaluations (95).

The evidence base itself places important limits on inference. Direct SSN reconstruction studies remain few and heterogeneous, and many clinical radiomics studies were designed to predict pathology rather than to test reconstruction-specific incremental value. Technical studies using solid nodules, nonpulmonary lesions, or phantoms establish sensitivity mechanisms but cannot establish SSN biological validity or patient benefit. Surgery-enriched cohorts further limit absolute-risk transportability to screening populations. Consequently, the strongest current conclusions concern image-domain dependence and technical feasibility, while evidence for calibrated incremental value and management benefit remains limited.

This review has methodological limitations. Evidence selection was purposeful rather than exhaustive, and the reported layer counts should be interpreted as a structured narrative-review evidence inventory rather than PRISMA-style study-flow estimates. No formal study-level risk-of-bias instrument or meta-analysis was applied. One author performed primary screening and two additional authors verified retained records and evidence-layer classification, with disagreements resolved by discussion. Heterogeneity in SSN definitions, acquisition and reconstruction protocols, segmentation, endpoints, validation, calibration, and reporting limits claims of completeness and precludes formal evidence-certainty grading.

Finally, the technical landscape continues to evolve as photon-counting CT, high-resolution detector systems, reconstruction algorithms, and newer AI models change the acquisition-to-biomarker relationship. Domain assumptions will therefore require periodic re-evaluation. The evidence layers, sensitivity map, use-case matrix, reporting items, and safeguards presented here are author-developed outputs from qualitative synthesis. They have not undergone external validation and are not certainty grades, consensus statements, reporting standards, or clinical guidelines.

## 8 Conclusions

CT reconstruction is part of the measurement domain for SSN radiomics because pixel size, section thickness, kernel, and reconstruction algorithm can alter segmentation, feature values, and model transportability. Reconstruction compatibility is therefore a model-applicability issue rather than a purely technical detail. Persistent pGGNs are a plausible setting for quantitative risk refinement, but greater benefit than in PSNs has not been demonstrated; for PSNs, any added value must exceed strong solid-component and longitudinal predictors.

Progress now depends on same-patient, preferably same-acquisition, reconstruction comparisons, externally calibrated multicenter validation, and prospective impact testing. Clinical use requires stable threshold performance, explicit domain checks, strong conventional comparators, and evidence that model-supported decisions improve care without avoidable harm. Until those requirements are met, reconstruction-aware radiomics should remain investigational or be used only within governed evaluation pathways rather than as an autonomous clinical pathway.
