# Supplementary material for: Reconstruction-aware radiomics for risk stratification of subsolid pulmonary nodules: technical evidence, clinical limits, and translation priorities
Source: Front Med (Lausanne). 2026 Oct 6;13:1955926. doi: 10.3389/fmed.2026.1955926 (PMC13681840; doi:10.3389/fmed.2026.1955926)
Supplement: Supplementary file 1 [file Datasheet1.pdf]

Supplementary Material

Supplementary Table S1. Modular literature-search framework and database-specific implementation.

Item	Logic / purpose	Terms or implementation
Search modules		
Module A	{SSN} AND {RECON}	Direct targeted/high-spatial-resolution SSN imaging and reconstruction-domain evidence.
Module B	{SSN} AND {AI} AND {OUTCOME}	Clinical SSN radiomics/quantitative-imaging studies addressing malignancy, invasiveness, persistence, growth, STAS, and related outcomes.
Module C	{BROAD} AND {AI} AND {TECH}	Technical robustness, reproducibility, reconstruction-domain variation, and harmonization evidence.
Reusable concept blocks		
{SSN}	SSN population	Controlled term where available; ground-glass, pure ground-glass, subsolid, and part-solid pulmonary nodule terms; GGN, pGGN, PSN; singular/plural variants.
{BROAD}	Broad lung/CT context	Controlled pulmonary-nodule/lung-neoplasm/CT terms where available; lung or pulmonary nodule*; ground-glass/subsolid nodule*; computed tomography; CT.
{RECON}	Acquisition/reconstruction	targeted/target reconstruction or scanning; small FOV; high/ultra-high spatial resolution; photon-counting CT; iterative/model-based/deep-learning reconstruction; reconstruction algorithm/kernel; section/slice thickness; interval; pixel/voxel/matrix size.
{AI}	Radiomics / quantitative imaging	radiomic*; texture analysis/features; quantitative imaging; image biomarker*; machine learning when linked to imaging or CT features.
{OUTCOME}	Clinical endpoints	malignan*; invasiv*; patholog*; persist*; growth; progression; spread through air spaces/STAS; predict*; classif*; risk.
{TECH}	Technical robustness	reconstruction/kernel; thickness; pixel/voxel size; dose/noise; resampling/interpolation; discretization; harmonization/ComBat/CovBat; reproducibility/repeatability/robustness/stability; scan-rescan/test-retest.
Database implementation		
PubMed/MEDLINE (NCBI)	MeSH plus free text	Supplementary Methods M1. Yields: A, n=94; B, n=188; C, n=712.
Embase.com	Emtree plus free text	Supplementary Methods M2. Yields: A, n=215; B, n=414; C, n=642.
Web of Science Core Collection	Topic/title fielded search	Supplementary Methods M3. Yields: A, n=119; B, n=231; C, n=513 (C1 OR C2).
Scopus	Title/abstract/keyword	Supplementary Methods M4. Yields: A, n=123; B, n=215; C, n=628.
Search date and reproducibility verification	Database search completed June 30, 2026; verification during revision	June 30, 2026 is the manuscript search-completion date. During revision, database-specific strings/exports were rechecked solely for reproducibility; this was not a new search/update and does not reconstruct every historical selection decision.
Structured evidence inventory	Primary evidence-layer assignment	Layer 1a, n=36; Layer 1b, n=19; Layer 2, n=236; Layer 3, n=167 (total n=458). These are structured evidence-inventory counts, not systematic-review inclusion totals.

Database searches across PubMed/MEDLINE, Embase, Web of Science Core Collection, and Scopus were completed on June 30, 2026 using three complementary modules. No language or document-type restrictions were applied. Concept blocks in Supplementary Table S1 summarize the search logic; exact database-specific syntax and term combinations are reproduced in Supplementary Methods M1-M4. Module-level counts are search yields and may overlap. Reproducibility verification during revision was not treated as a new literature-search or update date.

Supplementary Methods M1-M4. Final database-specific search strings and search yields.

The following strings document the final reproducible database-specific implementations of the three-module search framework. Module-level counts are search yields and may overlap across modules.

M1. PubMed/MEDLINE (NCBI)

Module A (n=94)

```
(
 "ground-glass nodule"[tiab]
 OR "ground-glass nodules"[tiab]
 OR "ground glass nodule"[tiab]
 OR "ground glass nodules"[tiab]
 OR "pure ground-glass nodule"[tiab]
 OR "pure ground-glass nodules"[tiab]
 OR "pure ground glass nodule"[tiab]
 OR "pure ground glass nodules"[tiab]
 OR "subsolid pulmonary nodule"[tiab]
 OR "subsolid pulmonary nodules"[tiab]
```

```
  OR "subsolid nodule"[tiab]
  OR "subsolid nodules"[tiab]
  OR "sub-solid nodule"[tiab]
  OR "sub-solid nodules"[tiab]
  OR "part-solid pulmonary nodule"[tiab]
  OR "part-solid pulmonary nodules"[tiab]
  OR "part-solid nodule"[tiab]
  OR "part-solid nodules"[tiab]
  OR "part solid nodule"[tiab]
  OR "part solid nodules"[tiab]
  OR "nonsolid nodule"[tiab]
  OR "nonsolid nodules"[tiab]
  OR "non-solid nodule"[tiab]
  OR "non-solid nodules"[tiab]
  OR "semisolid nodule"[Title/Abstract:~0]
  OR "semisolid nodules"[Title/Abstract:~0]
  OR "semi solid nodule"[Title/Abstract:~0]
  OR "semi solid nodules"[Title/Abstract:~0]
  OR GGN[tiab]
  OR GGNs[tiab]
  OR pGGN[tiab]
  OR pGGNs[tiab]
  OR PSN[tiab]
  OR PSNs[tiab]
)
AND
(
  "Tomography, X-Ray Computed"[Mesh]
  OR "computed tomography"[tiab]
  OR CT[tiab]
  OR "thin-section CT"[tiab]
  OR "thin section CT"[tiab]
  OR "high-resolution CT"[tiab]
  OR "high resolution CT"[tiab]
  OR HRCT[tiab]
)
AND
(
  "targeted reconstruction"[tiab]
  OR "target reconstruction"[tiab]
  OR "targeted scanning"[tiab]
  OR "target scanning"[tiab]
  OR "target scan"[tiab]
  OR "small field of view"[tiab]
  OR "small FOV"[tiab]
  OR "high spatial resolution"[tiab]
  OR "ultra-high-resolution CT"[tiab]
  OR "ultra high resolution CT"[tiab]
  OR UHRCT[tiab]
  OR "high-resolution reconstruction"[tiab]
  OR "iterative reconstruction"[tiab]
  OR "model-based iterative reconstruction"[tiab]
  OR "deep-learning reconstruction"[tiab]
  OR "deep learning reconstruction"[tiab]
  OR "photon-counting CT"[tiab]
  OR "photon counting CT"[tiab]
  OR "reconstruction algorithm"[tiab]
  OR "reconstruction algorithms"[tiab]
  OR "reconstruction kernel"[tiab]
  OR "reconstruction kernels"[tiab]
  OR "sharp kernel"[tiab]
  OR "sharp kernels"[tiab]
  OR "section thickness"[tiab]
  OR "slice thickness"[tiab]
  OR "pixel size"[tiab]
  OR "voxel size"[tiab]
```

```
  OR "matrix size"[tiab]
)
```

Module B (n=188)

```
(
  "ground-glass nodule"[tiab]
  OR "ground-glass nodules"[tiab]
  OR "ground glass nodule"[tiab]
  OR "ground glass nodules"[tiab]
  OR "pure ground-glass nodule"[tiab]
  OR "pure ground-glass nodules"[tiab]
  OR "pure ground glass nodule"[tiab]
  OR "pure ground glass nodules"[tiab]
  OR "subsolid pulmonary nodule"[tiab]
  OR "subsolid pulmonary nodules"[tiab]
  OR "subsolid nodule"[tiab]
  OR "subsolid nodules"[tiab]
  OR "sub-solid nodule"[tiab]
  OR "sub-solid nodules"[tiab]
  OR "part-solid nodule"[tiab]
  OR "part-solid nodules"[tiab]
  OR "part solid nodule"[tiab]
  OR "part solid nodules"[tiab]
  OR GGN[tiab]
  OR GGNs[tiab]
  OR pGGN[tiab]
  OR pGGNs[tiab]
  OR PSN[tiab]
  OR PSNs[tiab]
)
AND
(
  "Tomography, X-Ray Computed"[Mesh]
  OR "computed tomography"[tiab]
  OR CT[tiab]
  OR "thin-section CT"[tiab]
  OR "high-resolution CT"[tiab]
)
AND
(
  "Radiomics"[Mesh]
  OR radiomic*[tiab]
  OR "texture analysis"[tiab]
  OR "texture analyses"[tiab]
  OR "texture feature"[tiab]
  OR "texture features"[tiab]
  OR "quantitative imaging"[tiab]
  OR "image biomarker"[tiab]
  OR "image biomarkers"[tiab]
  OR
  (
    "machine learning"[tiab]
    AND
    (
      "imaging feature"[tiab]
      OR "imaging features"[tiab]
      OR "CT feature"[tiab]
      OR "CT features"[tiab]
    )
  )
)
AND
(
  malignan*[tiab]
  OR invasiv*[tiab]
  OR patholog*[tiab]
  OR persist*[tiab]
  OR growth[tiab]
```

```
  OR progression[tiab]
  OR "spread through air spaces"[tiab]
  OR STAS[tiab]
  OR predict*[tiab]
  OR classif*[tiab]
  OR risk[tiab]
)
```

Module C (n=712)

```
(
  "Tomography, X-Ray Computed"[Mesh]
  OR "computed tomography"[tiab]
  OR "CT imaging"[tiab]
  OR "CT image"[tiab]
  OR "CT images"[tiab]
)
AND
(
  "Radiomics"[Mesh]
  OR radiomic*[tiab]
  OR "texture analysis"[tiab]
  OR "texture analyses"[tiab]
  OR "texture feature"[tiab]
  OR "texture features"[tiab]
)
AND
(
  "reconstruction algorithm"[tiab]
  OR "reconstruction algorithms"[tiab]
  OR "reconstruction kernel"[tiab]
  OR "reconstruction kernels"[tiab]
  OR "section thickness"[tiab]
  OR "slice thickness"[tiab]
  OR "pixel size"[tiab]
  OR "voxel size"[tiab]
  OR "matrix size"[tiab]
  OR "field of view"[tiab]
  OR resampl*[tiab]
  OR interpolat*[tiab]
  OR discretiz*[tiab]
  OR discretis*[tiab]
  OR harmoniz*[tiab]
  OR harmonis*[tiab]
  OR ComBat[tiab]
  OR CovBat[tiab]
  OR "scan-rescan"[tiab]
  OR "scan rescan"[tiab]
  OR "test-retest"[tiab]
  OR "test retest"[tiab]
  OR
  (
    (
      reproducib*[tiab]
      OR repeatab*[tiab]
      OR robust*[tiab]
      OR stabil*[tiab]
    )
    AND
    (
      reconstruct*[tiab]
      OR acquisition[tiab]
      OR protocol*[tiab]
      OR preprocessing[tiab]
    )
  )
)
```

M2. Embase.com

Module A (n=215)

(
'ground glass nodule'/exp
OR 'ground glass nodule*':ti,ab,kw
OR 'ground-glass nodule*':ti,ab,kw
OR 'pure ground glass nodule*':ti,ab,kw
OR 'pure ground-glass nodule*':ti,ab,kw
OR 'subsolid pulmonary nodule*':ti,ab,kw
OR 'subsolid nodule*':ti,ab,kw
OR 'sub-solid nodule*':ti,ab,kw
OR 'part solid pulmonary nodule*':ti,ab,kw
OR 'part-solid pulmonary nodule*':ti,ab,kw
OR 'part solid nodule*':ti,ab,kw
OR 'part-solid nodule*':ti,ab,kw
OR 'nonsolid nodule*':ti,ab,kw
OR 'non-solid nodule*':ti,ab,kw
OR 'semisolid nodule*':ti,ab,kw
OR 'semi solid nodule*':ti,ab,kw
OR 'semi-solid nodule*':ti,ab,kw
OR ggn:ti,ab,kw
OR ggns:ti,ab,kw
OR pggn:ti,ab,kw
OR pggns:ti,ab,kw
OR psn:ti,ab,kw
OR psns:ti,ab,kw
)
AND
(
'computed tomography'/exp
OR 'computed tomography':ti,ab,kw
OR ct:ti,ab,kw
OR 'thin section ct':ti,ab,kw
OR 'thin-section ct':ti,ab,kw
OR 'high resolution ct':ti,ab,kw
OR 'high-resolution ct':ti,ab,kw
OR hrct:ti,ab,kw
)
AND
(
'targeted reconstruction':ti,ab,kw
OR 'target reconstruction':ti,ab,kw
OR 'targeted scanning':ti,ab,kw
OR 'target scanning':ti,ab,kw
OR 'target scan':ti,ab,kw
OR 'small field of view':ti,ab,kw
OR 'small fov':ti,ab,kw
OR 'high spatial resolution':ti,ab,kw
OR 'ultra high resolution ct':ti,ab,kw
OR 'ultra-high-resolution ct':ti,ab,kw
OR uhrct:ti,ab,kw
OR 'high resolution reconstruction':ti,ab,kw
OR 'high-resolution reconstruction':ti,ab,kw
OR 'iterative reconstruction':ti,ab,kw
OR 'model based iterative reconstruction':ti,ab,kw
OR 'model-based iterative reconstruction':ti,ab,kw
OR 'deep learning reconstruction':ti,ab,kw
OR 'deep-learning reconstruction':ti,ab,kw
OR 'photon counting ct':ti,ab,kw
OR 'photon-counting ct':ti,ab,kw
OR 'reconstruction algorithm*':ti,ab,kw
OR 'reconstruction kernel*':ti,ab,kw
OR 'sharp kernel*':ti,ab,kw
OR 'section thickness':ti,ab,kw
OR 'slice thickness':ti,ab,kw
OR 'pixel size':ti,ab,kw

OR 'voxel size':ti,ab,kw
OR 'matrix size':ti,ab,kw
)

Module B (n=414)

(
'ground glass nodule'/exp
OR 'ground glass nodule*':ti,ab,kw
OR 'ground-glass nodule*':ti,ab,kw
OR 'pure ground glass nodule*':ti,ab,kw
OR 'pure ground-glass nodule*':ti,ab,kw
OR 'subsolid pulmonary nodule*':ti,ab,kw
OR 'subsolid nodule*':ti,ab,kw
OR 'sub-solid nodule*':ti,ab,kw
OR 'part solid nodule*':ti,ab,kw
OR 'part-solid nodule*':ti,ab,kw
OR ggn:ti,ab,kw
OR ggns:ti,ab,kw
OR pggn:ti,ab,kw
OR pggns:ti,ab,kw
OR psn:ti,ab,kw
OR psns:ti,ab,kw
)
AND
(
'computed tomography'/exp
OR 'computed tomography':ti,ab,kw
OR ct:ti,ab,kw
OR 'thin section ct':ti,ab,kw
OR 'thin-section ct':ti,ab,kw
OR 'high resolution ct':ti,ab,kw
OR 'high-resolution ct':ti,ab,kw
)
AND
(
'radiomics'/exp
OR radiomic*:ti,ab,kw
OR 'texture analysis'/exp
OR 'texture analys*':ti,ab,kw
OR 'texture feature*':ti,ab,kw
OR 'quantitative imaging':ti,ab,kw
OR 'image biomarker*':ti,ab,kw
OR
(
'machine learning':ti,ab,kw
AND
(
'imaging feature*':ti,ab,kw
OR 'ct feature*':ti,ab,kw
)
)
)
AND
(
malignan*:ti,ab,kw
OR invasiv*:ti,ab,kw
OR patholog*:ti,ab,kw
OR persist*:ti,ab,kw
OR growth:ti,ab,kw
OR progression:ti,ab,kw
OR 'spread through air spaces':ti,ab,kw
OR stas:ti,ab,kw
OR predict*:ti,ab,kw
OR classif*:ti,ab,kw
OR risk:ti,ab,kw
)

Module C (n=642)

```
(
  'computed tomography':ti,ab
  OR 'ct imaging':ti,ab
  OR 'ct image*':ti,ab
  OR ct:ti
)
AND
(
  'radiomics'/exp
  OR radiomic*:ti,ab
  OR 'texture analys*':ti,ab
  OR 'texture feature*':ti,ab
)
AND
(
  'reconstruction algorithm*':ti,ab
  OR 'reconstruction kernel*':ti,ab
  OR 'reconstruction parameter*':ti,ab
  OR 'reconstruction setting*':ti,ab
  OR 'reconstruction technique*':ti,ab
  OR 'acquisition parameter*':ti,ab
  OR 'section thickness':ti,ab
  OR 'slice thickness':ti,ab
  OR 'pixel size':ti,ab
  OR 'voxel size':ti,ab
  OR 'matrix size':ti,ab
  OR 'field of view':ti,ab
  OR 'scanner variation*':ti,ab
  OR 'inter scanner':ti,ab
  OR interscanner:ti,ab
  OR resampl*:ti,ab
  OR interpolat*:ti,ab
  OR discretiz*:ti,ab
  OR discretis*:ti,ab
  OR binning:ti,ab
  OR harmoniz*:ti,ab
  OR harmonis*:ti,ab
  OR combat:ti,ab
  OR covbat:ti,ab
  OR 'scan rescan':ti,ab
  OR 'scan-rescan':ti,ab
  OR 'test retest':ti,ab
  OR 'test-retest':ti,ab
)
AND
(
  reproducib*:ti,ab
  OR repeatab*:ti,ab
  OR robust*:ti,ab
  OR stabil*:ti,ab
  OR variab*:ti,ab
  OR 'feature drift':ti,ab
  OR 'domain shift':ti,ab
  OR sensitivity:ti,ab
)
```

M3. Web of Science Core Collection (Editions: All)

Module A (n=119)

```
TS=(
"ground-glass nodule*" OR
"ground glass nodule*" OR
"pure ground-glass nodule*" OR
"pure ground glass nodule*" OR
"subsolid pulmonary nodule*" OR
"subsolid nodule*" OR
"sub-solid nodule*" OR
```

"part-solid pulmonary nodule*" OR
"part solid pulmonary nodule*" OR
"part-solid nodule*" OR
"part solid nodule*" OR
"nonsolid nodule*" OR
"non-solid nodule*" OR
"semisolid nodule*" OR
"semi-solid nodule*" OR
"semi solid nodule*" OR
GGN OR GGNs OR
pGGN OR pGGNs OR
PSN OR PSNs
)
AND TS=(
"computed tomography" OR
CT OR
"thin-section CT" OR
"thin section CT" OR
"high-resolution CT" OR
"high resolution CT" OR
HRCT
)
AND TS=(
"targeted reconstruction" OR
"target reconstruction" OR
"targeted scanning" OR
"target scanning" OR
"target scan" OR
"small field of view" OR
"small FOV" OR
"high spatial resolution" OR
"ultra-high-resolution CT" OR
"ultra high resolution CT" OR
UHRCT OR
"high-resolution reconstruction" OR
"high resolution reconstruction" OR
"iterative reconstruction" OR
"model-based iterative reconstruction" OR
"model based iterative reconstruction" OR
"deep-learning reconstruction" OR
"deep learning reconstruction" OR
"photon-counting CT" OR
"photon counting CT" OR
"reconstruction algorithm*" OR
"reconstruction kernel*" OR
"sharp kernel*" OR
"section thickness" OR
"slice thickness" OR
"pixel size" OR
"voxel size" OR
"matrix size"
)

Module B (n=231)

TS=(
"ground-glass nodule*" OR
"ground glass nodule*" OR
"pure ground-glass nodule*" OR
"pure ground glass nodule*" OR
"subsolid pulmonary nodule*" OR
"subsolid nodule*" OR
"sub-solid nodule*" OR
"part-solid nodule*" OR
"part solid nodule*" OR
GGN OR GGNs OR
pGGN OR pGGNs OR
PSN OR PSNs
)

```
AND TS=(
"computed tomography" OR
CT OR
"thin-section CT" OR
"thin section CT" OR
"high-resolution CT" OR
"high resolution CT"
)
AND TS=(
radiomic* OR
"texture analys*" OR
"texture feature*" OR
"quantitative imaging" OR
"image biomarker*" OR
(
  "machine learning"
  AND
  (
    "imaging feature*" OR
    "CT feature*"
  )
)
)
AND TS=(
malignan* OR
invasiv* OR
patholog* OR
persist* OR
growth OR
progression OR
"spread through air spaces" OR
STAS OR
predict* OR
classif* OR
risk
)
```

Module C1

```
TS=(
"computed tomography" OR
"CT imaging" OR
"CT image*"
)
AND TS=(
radiomic* OR
"texture analys*" OR
"texture feature*"
)
AND TS=(
"reconstruction algorithm*" OR
"reconstruction kernel*" OR
"reconstruction parameter*" OR
"reconstruction setting*" OR
"reconstruction technique*" OR
"acquisition parameter*" OR
"section thickness" OR
"slice thickness" OR
"pixel size" OR
"voxel size" OR
"matrix size" OR
"field of view" OR
"scanner variation*" OR
"inter scanner" OR
interscanner OR
resampl* OR
interpolat* OR
discretiz* OR
discretis* OR
```

binning OR
harmoniz* OR
harmonis* OR
ComBat OR
CovBat OR
"scan rescan" OR
"scan-rescan" OR
"test retest" OR
"test-retest"
)
AND TS=(
reproducib* OR
repeatab* OR
robust* OR
stabil* OR
variab* OR
"feature drift" OR
"domain shift" OR
sensitivity
)

Module C2

TI=(CT)
AND TS=(
radiomic* OR
"texture analys*" OR
"texture feature*"
)
AND TS=(
"reconstruction algorithm*" OR
"reconstruction kernel*" OR
"reconstruction parameter*" OR
"reconstruction setting*" OR
"reconstruction technique*" OR
"acquisition parameter*" OR
"section thickness" OR
"slice thickness" OR
"pixel size" OR
"voxel size" OR
"matrix size" OR
"field of view" OR
"scanner variation*" OR
"inter scanner" OR
interscanner OR
resampl* OR
interpolat* OR
discretiz* OR
discretis* OR
binning OR
harmoniz* OR
harmonis* OR
ComBat OR
CovBat OR
"scan rescan" OR
"scan-rescan" OR
"test retest" OR
"test-retest"
)
AND TS=(
reproducib* OR
repeatab* OR
robust* OR
stabil* OR
variab* OR
"feature drift" OR
"domain shift" OR
sensitivity
)

Module C final: C1 OR C2, n=513.

M4. Scopus

Module A (n=123)

```
TITLE-ABS-KEY(
 (
  "ground-glass nodule*" OR
  "ground glass nodule*" OR
  "pure ground-glass nodule*" OR
  "pure ground glass nodule*" OR
  "subsolid pulmonary nodule*" OR
  "subsolid nodule*" OR
  "sub-solid nodule*" OR
  "part-solid pulmonary nodule*" OR
  "part solid pulmonary nodule*" OR
  "part-solid nodule*" OR
  "part solid nodule*" OR
  "nonsolid nodule*" OR
  "non-solid nodule*" OR
  "semisolid nodule*" OR
  "semi-solid nodule*" OR
  "semi solid nodule*" OR
  GGN OR GGNs OR
  pGGN OR pGGNs OR
  PSN OR PSNs
 )
 AND
 (
  "computed tomography" OR
  CT OR
  "thin-section CT" OR
  "thin section CT" OR
  "high-resolution CT" OR
  "high resolution CT" OR
  HRCT
 )
 AND
 (
  "targeted reconstruction" OR
  "target reconstruction" OR
  "targeted scanning" OR
  "target scanning" OR
  "target scan" OR
  "small field of view" OR
  "small FOV" OR
  "high spatial resolution" OR
  "ultra-high-resolution CT" OR
  "ultra high resolution CT" OR
  UHRCT OR
  "high-resolution reconstruction" OR
  "high resolution reconstruction" OR
  "iterative reconstruction" OR
  "model-based iterative reconstruction" OR
  "model based iterative reconstruction" OR
  "deep-learning reconstruction" OR
  "deep learning reconstruction" OR
  "photon-counting CT" OR
  "photon counting CT" OR
  "reconstruction algorithm*" OR
  "reconstruction kernel*" OR
  "sharp kernel*" OR
  "section thickness" OR
  "slice thickness" OR
  "pixel size" OR
  "voxel size" OR
  "matrix size"
```

```
 )
)
```

Module B (n=215)

```
TITLE-ABS-KEY(
 (
  "ground-glass nodule*" OR
  "ground glass nodule*" OR
  "pure ground-glass nodule*" OR
  "pure ground glass nodule*" OR
  "subsolid pulmonary nodule*" OR
  "subsolid nodule*" OR
  "sub-solid nodule*" OR
  "part-solid nodule*" OR
  "part solid nodule*" OR
  GGN OR GGNs OR
  pGGN OR pGGNs OR
  PSN OR PSNs
 )
 AND
 (
  "computed tomography" OR
  CT OR
  "thin-section CT" OR
  "thin section CT" OR
  "high-resolution CT" OR
  "high resolution CT"
 )
 AND
 (
  radiomic* OR
  "texture analys*" OR
  "texture feature*" OR
  "quantitative imaging" OR
  "image biomarker*" OR
  (
   "machine learning"
   AND
   (
    "imaging feature*" OR
    "CT feature*"
   )
  )
 )
 AND
 (
  malignan* OR
  invasiv* OR
  patholog* OR
  persist* OR
  growth OR
  progression OR
  "spread through air spaces" OR
  STAS OR
  predict* OR
  classif* OR
  risk
 )
)
```

Module C (n=628)

```
(
 TITLE-ABS-KEY(
  "computed tomography" OR
  "CT imaging" OR
  "CT image*"
 )
 OR
```

```
  TITLE(CT)
)
AND
TITLE-ABS-KEY(
  radiomic* OR
  "texture analys*" OR
  "texture feature*"
)
AND
TITLE-ABS-KEY(
  "reconstruction algorithm*" OR
  "reconstruction kernel*" OR
  "reconstruction parameter*" OR
  "reconstruction setting*" OR
  "reconstruction technique*" OR
  "acquisition parameter*" OR
  "section thickness" OR
  "slice thickness" OR
  "pixel size" OR
  "voxel size" OR
  "matrix size" OR
  "field of view" OR
  "scanner variation*" OR
  "inter scanner" OR
  interscanner OR
  resampl* OR
  interpolat* OR
  discretiz* OR
  discretis* OR
  binning OR
  harmoniz* OR
  harmonis* OR
  ComBat OR
  CovBat OR
  "scan rescan" OR
  "scan-rescan" OR
  "test retest" OR
  "test-retest"
)
AND
TITLE-ABS-KEY(
  reproducib* OR
  repeatab* OR
  robust* OR
  stabil* OR
  variab* OR
  "feature drift" OR
  "domain shift" OR
  sensitivity
)
```

Supplementary Table S2. SANRA-informed methodological self-assessment.

SANRA domain	How addressed	Residual limitation
Justification of importance	Introduction defines the management problem and reconstruction-sensitive uncertainty in SSNs.	Narrative emphasis; no formal burden-of-disease synthesis.
Statement of aims	Methods define SSN populations, task-specific endpoints, scope, evidence layers, and comparative-reconstruction terminology.	Purposeful rather than exhaustive; evidence-layer proximity is not quality or certainty.
Description of literature searching	Methods, Table S1, and Methods M1-M4 report four databases, June 30, 2026 completion date, exact queries/yields, eligibility principles, screening workflow, disagreement resolution, and the structured evidence inventory.	A complete record-level screening/exclusion log was not prospectively retained; reproducibility verification during revision cannot reconstruct every historical selection decision.
Referencing	Claims are linked to direct SSN, technical-robustness, and clinical-application evidence; Table 4 gives representative studies and Table S4 expanded standardized extraction.	Not every potentially eligible publication was catalogued.
Scientific reasoning	Synthesis separates image depiction, domain stability, predictive validity, incremental value, management impact, and economic implications, while distinguishing direct from indirect evidence.	Evidence layers and other review outputs are author-developed qualitative structures without external validation.
Presentation of endpoint data	Tables 1-4 and S3-S4 distinguish tasks, subtype-specific implications, comparators, calibration, error costs, limitations, safeguards, and translational domains.	No quantitative pooling because tasks and study designs were heterogeneous.

SANRA (20) informed methodological self-assessment only; it was not used as a quality certification, formal compliance claim, study-level risk-of-bias tool, or evidence-certainty framework. The evidence layers and other author-developed outputs have not undergone external validation. SANRA, Scale for the Assessment of Narrative Review Articles.

Supplementary Table S3. Proposed reconstruction-domain reporting items for subsolid pulmonary nodule radiomics.

Domain	Report	Rationale	Primary evidence basis
Clinical task, SSN subtype, and lung context	Specify pGGN/PSN; newly detected, persistent, longitudinal, multifocal, or preoperative setting; intended user; endpoint/reference standard; linked action; relevant emphysema or fibrosing ILD in the intended-use population.	Tasks/error costs differ by subtype and context; multifocality and structural lung disease can affect interpretation and transportability.	Mainly Layer 3; contextual clinical evidence and author-developed applicability considerations
Scanner and acquisition	Manufacturer/model; detector configuration; acquisition mode; kVp; mAs or CTDIvol; pitch; collimation; spectral/energy setting if applicable.	Sampling, dose, detector mode, and energy setting affect spatial resolution, contrast, and noise texture.	Layers 1a, 1b, and 2
Reconstruction geometry	Section thickness/interval; reconstructed FOV; matrix; in-plane pixel dimensions.	Voxel geometry affects partial volume, segmentation, conventional measurements, and radiomic values.	Layers 1a, 1b, and 2
Kernel and algorithm	Kernel name/strength; filtered back projection, iterative/model-based/deep-learning method; version/level; spectral or monoenergetic reconstruction if applicable.	Edge sharpness, noise texture, smoothing, and spectral characteristics are reconstruction-dependent.	Layers 1a, 1b, and 2
Targeted technique	State whether new projection data were acquired or existing raw data reconstructed; relation to original acquisition; added radiation if any.	Distinguishes targeted scanning from targeted reconstruction and defines acquisition constraints.	Layers 1a and 1b
Preprocessing	Resampling target/interpolation; HU range; discretization method/value; intensity normalization if used; filter parameters.	Preprocessing can introduce feature drift and cannot restore information lost upstream.	Layer 2
Segmentation and quality control	pGGN/PSN-specific ROI strategy; whole-lesion/regional definitions; reader or algorithm; software/version; agreement/robustness testing; failure criteria; handling of vessels, pleura, and airways.	Small, low-contrast SSNs are vulnerable to boundary, regional-definition, and segmentation instability.	Layers 1a, 1b, and 2
Harmonization	Method/software version; covariates; fitting set; training-only implementation; leakage control; sensitivity analysis.	Cannot recover lost spatial information and may remove biological signal or mask domain-specific failure.	Layer 2
Cross-domain validation and comparators	Prefer paired same-patient, ideally same-acquisition, reconstructions; patient-level partitions; strong guideline/expert/conventional comparators; report discrimination, calibration, Brier score, net benefit, and threshold stability.	Internal AUC alone cannot establish reconstruction-specific incremental value or threshold transportability.	Layers 1a, 2, and 3
Deployment compatibility	Supported scanners/domains; automated domain check; local recalibration; prespecified thresholds; abstention, updating, version control, and monitoring.	Internal performance or visual improvement does not establish transportability, threshold validity, or safety.	Mainly Layer 3

These evidence-informed, author-proposed reporting items synthesize direct SSN evidence, indirect technical-robustness evidence, established radiomics/prediction-model guidance, and clinical-context considerations (14,47,59,60,62,63). Multifocality and structural lung disease define applicability testing rather than established reconstruction-aware evidence. The items complement, rather than replace, existing guidance and are not an externally validated minimum standard. Evidence layers indicate primary evidentiary basis, not methodological quality or certainty. Abbreviations: AUC, area under the receiver operating characteristic curve; CTR, consolidation-to-tumor ratio; CTDIvol, volume CT dose index; FOV, field of view; HU, Hounsfield unit; pGGN, pure ground-glass nodule; PSN, part-solid nodule; ROI, region of interest; SSN, subsolid pulmonary nodule.

Supplementary Table S4. Standardized extraction of representative evidence by reconstruction domain, validation, calibration, comparator, endpoint, and translational domain.

Study	Evidence source / design	Reconstruction or technical exposure	Validation / calibration / comparator	Endpoint and translational interpretation
Zhu et al. 2019 (27)	Direct SSN reconstruction evidence; Layer 1a; comparative imaging-domain study.	Ultra-high-resolution target scan vs conventional target reconstruction; acquisition and reconstruction changed together, so the reconstruction effect was not isolated.	Validation: pathology-category agreement. Calibration/external validation: NR*. Comparator: conventional target reconstruction for morphology.	Fine-structure depiction/pathology-category agreement; highest supported domain: image depiction/measurement. Does not establish radiomic robustness, reconstruction-specific incremental prediction, or management benefit.
Ren et al. 2021 (28)	Direct SSN evidence; Layer 1b; noncomparative high-resolution study.	Dedicated ultra-high-resolution target scanning; no paired standard-domain comparator.	Reference standard: pathology. External validation/calibration: NR*. Paired standard-reconstruction comparator: NR*.	Preinvasive vs invasive pathology using high-resolution morphologic/attenuation variables; supports association/discrimination within that domain. Reconstruction-specific incremental value remains unproven.
Tao et al. 2021 (31)	Direct GGN reconstruction-domain evidence; Layers 1a/2; same-scan pixel-size comparison.	Pixel-size domains compared within the same CT examination; 87% of 788 features varied and selected signatures changed.	Internal retrospective evaluation. External validation/calibration: NR*. Comparator: pixel-size domains. Locked action threshold: NR*.	Supports feature robustness/domain dependence and internal invasiveness discrimination. Does not prove universal superiority or patient benefit.
Meyer et al. 2019 (33)	Indirect non-SSN technical evidence; Layer 2; within-patient reconstruction study.	78 patients/151 liver metastases; 28 reconstructions per patient; dose/reconstruction settings varied; 11% of 106 features were reproducible across all variations.	Technical reproducibility endpoint. Calibration: N/A. Clinical comparator/SSN external validation: none.	Supports feature repeatability/robustness; applicability to SSN biology, segmentation, and management is indirect.
Emaminejad et al. 2021 (34)	Indirect pulmonary-nodule technical evidence; Layer 2; multivariable parameter study.	89 screening patients with solid nodules >4 mm; 36 dose/thickness/kernel conditions; simulated dose reduction.	Technical reproducibility across parameter combinations. Calibration: N/A. Clinical comparator/SSN validation: none.	Supports parameter interactions and feature repeatability/robustness; does not establish SSN biological validity or clinical utility.

Study	Evidence source / design	Reconstruction or technical exposure	Validation / calibration / comparator	Endpoint and translational interpretation
Hertel et al. 2023 (36)	Indirect phantom technical evidence; Layer 2; photon-counting CT test-retest study.	Organic phantoms; 104 features; fixed-position test-retest, repositioning, and dose comparisons; stability 70% fixed and 65% repositioned.	Phantom test-retest validation. Calibration: N/A. Clinical comparator/patient-SSN validation: none.	Supports technical repeatability under defined conditions; cannot establish SSN biology, segmentation validity, or clinical benefit.
Wu et al. 2020 (79)	Clinical SSN evidence not reconstruction-specific; Layer 3; multicenter PSN prediction.	Regional solid/ground-glass radiomics; no targeted-reconstruction comparison.	Held-out test set; calibration reported as good. Comparators: Brock, clinical-semantic, volumetric, and whole-lesion models.	Supports discrimination, calibration, and incremental performance in PSNs; does not establish reconstruction-specific gain or prospective management benefit.
Wu et al. 2021 (46)	Clinical GGN evidence not reconstruction-specific; Layer 3; regional/peritumoral radiomics.	121 GGN adenocarcinomas; 2-mm vs 5-mm peritumoral regions; no reconstruction-domain comparison.	Single-center internal validation; external validation: none; calibration: NR*. Comparator: 2-mm vs 5-mm regions; no universal margin established.	Supports internal invasiveness discrimination/feature-region feasibility; incremental clinical and reconstruction-specific value remain unproven.
Xue et al. 2024 (83)	Clinical longitudinal GGN evidence not reconstruction-specific; Layer 3; delta-radiomics.	303 resected GGNs; baseline/follow-up CT; heterogeneous scanners/matrices; no controlled reconstruction-domain comparison.	Internal 7:3 split; external validation: none; calibration: NR*. Comparator: conventional radiomics-clinical model; delta-radiomics did not significantly outperform it.	Supports longitudinal internal discrimination; no clear incremental gain from delta-radiomics in this cohort.
Bao et al. 2025 (86)	Clinical SSN-related evidence not reconstruction-specific; Layer 3; multicenter STAS prediction.	Three-center retrospective surgical cohort; intratumoral, peritumoral, and clinical modeling; no reconstruction-domain comparison.	External test cohort from two centers; calibration and decision-curve analysis reported. Detailed conventional comparator: NR*.	Supports external discrimination, calibration, and decision-curve evaluation for a pathology surrogate; prospective surgical/patient benefit remains untested.

Evidence-source categories indicate what each study directly establishes. Translational domains progress from image depiction/measurement to feature robustness, discrimination, calibration/threshold performance, incremental value, and prospective management/patient benefit; support at one domain does not establish the next. NR* = not reported in the current review extraction and does not prove absence from the original article. N/A = not applicable to the stated technical endpoint. Evidence layers describe function and proximity, not methodological quality or certainty.

Supplementary Table S5. Evidence basis for the conceptual radiomic feature-family sensitivity map in Figure 3.

Feature family	Pixel size	Section thickness	Reconstruction kernel	Gray-level discretization	Image noise	Interpolation / resampling	Primary evidence basis
Shape	**M**	**M**	**M**	**N/A**	**M**	**M**	Direct GGN pixel-size evidence and broader CT robustness studies; segmentation and voxel-geometry dependence are recurrent themes (17,31–34,47).
First-order	**M**	**M**	**M**	**M**	**M**	**M**	CT robustness studies show dependence on voxel geometry, reconstruction, dose/noise, and intensity handling; IBSI provides standardized definitions (17,31–34,47).
GLCM	**H**	**H**	**H**	**M**	**H**	**H**	Texture dependence on pixel/voxel geometry, kernel, noise, and preprocessing is repeatedly reported; discretization effects are variable across implementations (17,31–34,45,47).
GLRLM	**H**	**H**	**H**	**H**	**H**	**H**	Run-length texture features have shown sensitivity to reconstruction and preprocessing choices across broader CT radiomics robustness studies (17,32–34,45,47).
GLSZM	**H**	**H**	**H**	**H**	**H**	**H**	Size-zone features have shown parameter dependence across voxel geometry, reconstruction, gray-level handling, and resampling settings in broader technical studies (17,32–34,45,47).
GLDM	**H**	**H**	**H**	**H**	**H**	**H**	The H assignments reflect repeated reports of technical dependence in broader radiomics robustness and standardization studies; direct SSN-specific evidence remains limited (17,32–34,45,47).
NGTDM	**H**	**H**	**H**	**H**	**H**	**H**	Neighborhood gray-tone features have shown sensitivity to image-domain and preprocessing changes in technical radiomics studies; direct SSN-specific evidence remains limited (17,32–34,45,47).
Wavelet / LoG	**U**	**U**	**H**	**U**	**H**	**H**	Filtered features introduce additional kernel/filter and interpolation dependence; evidence for pixel size, thickness, and discretization is less consistent, so these cells remain U (17,33,34,45).

Note. M = recurrent but moderate or context-dependent sensitivity; H = repeatedly reported and comparatively prominent dependence; U = insufficient, sparse, or inconsistent evidence; N/A = not directly applicable under the stated feature definition. These author-developed assignments are qualitative synthesis categories, not pooled effect estimates, numerical thresholds, or formal certainty grades. They are not invariant across scanners, protocols, segmentation methods, or software. Direct SSN-specific evidence is limited for several cells; broader pulmonary-nodule, nonpulmonary, and technical radiomics evidence supports technical sensitivity only, not SSN biological validity or clinical utility. GLCM, gray-level co-occurrence matrix; GLRLM, gray-level run-length matrix; GLSZM, gray-level size-zone matrix; GLDM, gray-level dependence matrix; NGTDM, neighborhood gray-tone difference matrix; LoG, Laplacian-of-Gaussian.
